# Abiotic Stresses Modulate Landscape of Poplar Transcriptome via Alternative Splicing, Differential Intron Retention, and Isoform Ratio Switching

**DOI:** 10.3389/fpls.2018.00005

**Published:** 2018-02-12

**Authors:** Sergei A. Filichkin, Michael Hamilton, Palitha D. Dharmawardhana, Sunil K. Singh, Christopher Sullivan, Asa Ben-Hur, Anireddy S. N. Reddy, Pankaj Jaiswal

**Affiliations:** ^1^Department of Botany and Plant Pathology, Oregon State University, Corvallis, OR, United States; ^2^Department of Computer Science, Colorado State University, Fort Collins, CO, United States; ^3^Center for Genome Research and Biocomputing, Oregon State University, Corvallis, OR, United States; ^4^Department of Biology and Program in Cell and Molecular Biology, Colorado State University, Fort Collins, CO, United States

**Keywords:** western poplar, transcriptome, alternative splicing, abiotic stress, isoform switching, differential intron retention, stress adaptation

## Abstract

Abiotic stresses affect plant physiology, development, growth, and alter pre-mRNA splicing. Western poplar is a model woody tree and a potential bioenergy feedstock. To investigate the extent of stress-regulated alternative splicing (AS), we conducted an in-depth survey of leaf, root, and stem xylem transcriptomes under drought, salt, or temperature stress. Analysis of approximately one billion of genome-aligned RNA-Seq reads from tissue- or stress-specific libraries revealed over fifteen millions of novel splice junctions. Transcript models supported by both RNA-Seq and single molecule isoform sequencing (Iso-Seq) data revealed a broad array of novel stress- and/or tissue-specific isoforms. Analysis of Iso-Seq data also resulted in the discovery of 15,087 novel transcribed regions of which 164 show AS. Our findings demonstrate that abiotic stresses profoundly perturb transcript isoform profiles and trigger widespread intron retention (IR) events. Stress treatments often increased or decreased retention of specific introns – a phenomenon described here as differential intron retention (DIR). Many differentially retained introns were regulated in a stress- and/or tissue-specific manner. A subset of transcripts harboring super stress-responsive DIR events showed persisting fluctuations in the degree of IR across all treatments and tissue types. To investigate coordinated dynamics of intron-containing transcripts in the study we quantified absolute copy number of isoforms of two conserved transcription factors (TFs) using Droplet Digital PCR. This case study suggests that stress treatments can be associated with coordinated switches in relative ratios between fully spliced and intron-retaining isoforms and may play a role in adjusting transcriptome to abiotic stresses.

## Introduction

**FIGURE 1:**
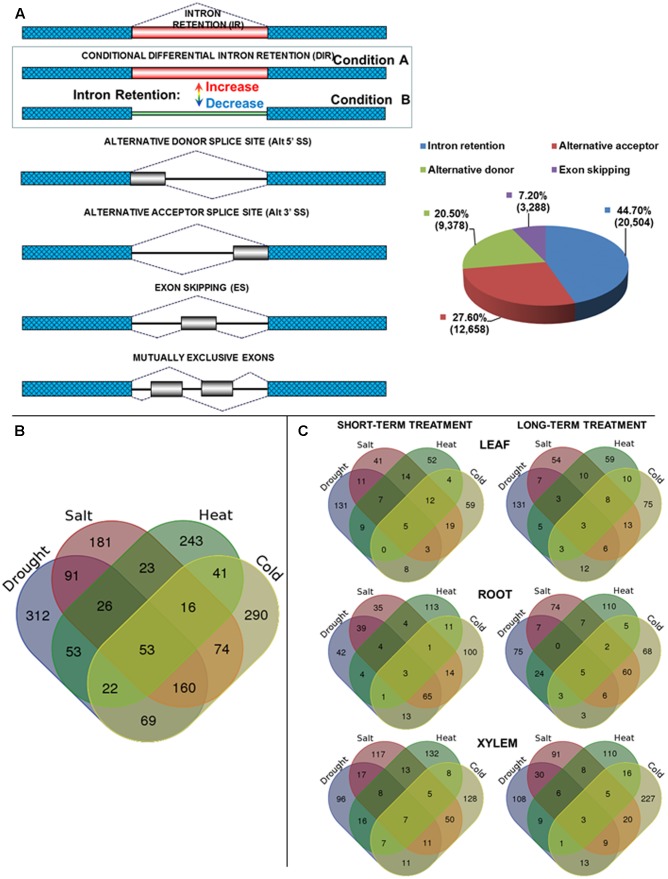
**(A)** Major classes of alternative splicing (AS) events. Differential intron retention (DIR) is a particular type of the intron retention events characterized by condition-dependent increase or decrease in the degree of retention of certain introns. The pie chart (right) shows distribution of major AS types in *Populus trichocarpa* transcriptome as estimated from Iso-Seq data. Mutually exclusive exons were classified as a subcategory of more general ES class. **(B)** Distribution of genes that encode transcripts harboring stress-regulated differentially retained introns. Venn diagram illustrating intersection between the gene loci associated with stress-regulated DIR events. Lists of DIR-harboring genes were combined across all tissue types. Both short term and prolonged treatments were combined for each stress type. **(C)** Venn diagram showing intersection between DIR-associated genes during the short or prolonged phases of each stress treatment within same tissue type.

Alternative splicing (AS) can increase a complexity of transcriptome and proteome via generating multiple transcripts and protein isoforms from the single gene. Up to 95% of mammalian precursor messenger RNAs (pre-mRNAs) are alternatively spliced (Pan et al., 2008) whereas recent studies in higher plants suggest that AS is fairly conserved (Mei et al., 2017a) and is observed in approximately 42–61% of intron-containing genes (Filichkin et al., 2010; Marquez et al., 2012). Common types of AS include intron retention (IR), exon skipping (ES), the alternative donor (alt 5′) or acceptor (alt 3′) splice site, and mutually exclusive exons (**Figure 1A**). IR is the prevalent mode of AS in plants and represents at least ∼40% of all AS events (Filichkin et al., 2010; Marquez et al., 2012; Shen et al., 2014) whereas ES is considered a predominant mode in animals (Pan et al., 2008). AS resulting in alternative open reading frames may increase proteome diversity. Increasing evidence suggests that AS is involved in regulation of development and cellular responses to environmental stresses in plants (Wang and Brendel, 2006; Barbazuk et al., 2008; Filichkin et al., 2010, 2015a; Vitulo et al., 2014; Li et al., 2016; Mei et al., 2017b). High salinity stress affects AS and IR of ∼49% of all intron-containing *Arabidopsis* genes (Ding et al., 2014). Heat stress triggers widespread IR including specific events in the first line response factors such as heat shock transcription factors (TFs) (Filichkin et al., 2010; Jiang et al., 2017). Drought is another common stress that profoundly perturbs AS patterns. Conversely, a single aberrant ES event in the *Arabidopsis* mRNA encoding Δ*1-pyrroline-5-carboxylate synthase1* reduced plant drought tolerance (Kesari et al., 2012).

Both full IR and alternative donor (Alt 5′) and acceptor (Alt 3′) events often introduce premature termination codons (PTCs) and trigger nonsense-mediated mRNA decay (NMD) in mammals (Maquat, 2005), flies (Nicholson and Muhlemann, 2010), and worms (Casadio et al., 2015). However, despite the presence of such NMD-eliciting features, the majority of *Arabidopsis* transcripts with full IR escape the NMD pathway (Kalyna et al., 2012). In contrast, many Alt 5′ and Alt 3′ events can elicit NMD response (Filichkin and Mockler, 2012; Kalyna et al., 2012; Staiger and Brown, 2013; Filichkin et al., 2015b). One possible explanation of NMD-insensitivity of the full intron-retaining mRNAs is their reversible sequestration (Boothby and Wolniak, 2011; Boothby et al., 2013; Filichkin et al., 2015a). Indeed, Boothby et al. (2013) showed that translation of such masked mRNAs in the male gametophyte of the fern *Marsilea vestita* activated upon introns removal.

In contrast to plants, ES is a predominant type of AS in mammalian cells. However, recent close examination of high coverage RNA-Seq data suggested that IR can affect as many as three-quarters of all mammalian genes with multiple exons (Braunschweig et al., 2014). Significantly, widespread IR is a signature feature of many human cancers (Dvinge and Bradley, 2015) and plays a role in the mechanism of tumor-suppressor inactivation (Jung and Lee, 2015). Even though IR is a prevailing mode of AS in higher plants its functional role(s) during stress adaptation remain unclear.

Western poplar (*Populus trichocarpa*) is a model woody plant and a commercial crop with a potential for bioenergy production. *Populus* species from different geographical zones display remarkably flexible adaptation to diverse environmental conditions (Soolanayakanahally et al., 2015). Increasing evidence suggests that AS is widespread and diversified across *Populus* species and could potentially contribute to the environmental adaptation, development of specialized tissues such as secondary xylem (wood) (Bao et al., 2013). Up to 25% of AS events in poplar (Xu et al., 2014) are estimated to generate protein domain modifications and therefore may contribute to proteome complexity during adaptation to the stresses. However, a comprehensive analysis of global changes in AS events across all main poplar tissues types induced by common abiotic stresses is currently unavailable. Here, we conducted a high resolution survey of AS and IR events in leaf, stem xylem, and root tissues under drought, salt, and temperature stresses. Stress-induced statistically significant IR rates were calculated across all detected IR events. We found that differential intron retention (DIR) is a widespread and often tissue- and/or stress-specific phenomenon in poplar. We identified subsets of transcripts showing similar dynamics of differential IR. Case study of two conserved eukaryotic TFs showed that relative ratios of DIR-harboring isoforms decrease during stress-induced up-regulation of their fully spliced mRNAs and vice versa. Analyses of both RNA-Seq and Iso-Seq (single molecule real time isoform sequencing) datasets generated thousands of novel protein-coding and non-coding gene models including those encoding for stress- and/or tissue-specific transcripts.

## Materials and Methods

### Plant Materials and Growth Conditions

Approximately 3 months old plants of Western poplar (*P. trichocarpa*, clone Nisqually 1) were propagated from stem cuttings and grown in 2 L pots at 12 h day/12 h night photo cycles with light intensity 300 μmoles/m^2^/s. For heat stress, plants were treated at 39°C for 12 h (short term) or 7 days (prolonged). For cold stress plants were subjected to 4°C (night) and 12°C (day) for 24 h (short term) or 7 days (prolonged). For drought treatment watering was withheld until the moisture of soil reached 0.1 m^3^/m^3^ and maintained at the level of 0.06 – 0.1 m^3^/m^3^. For a short term drought, stress plants were grown for 5 days after water withholding (an initial leaf wilting point) or for 7 days after initial leaf wilting (prolonged treatment, 12 days after withholding water). For high salinity, stress plants were treated with 100 mM sodium chloride solution for 24 h (short term) or for 7 days (prolonged). Root, leaf, and xylem tissues from both short- and long-term treatments were collected each in three independent biological replicates (**Supplementary File 1**). Controls represented the same tissue types of untreated plants. For the high resolution heat-cold stress time course plants were subjected to the 24 h heat treatment (42°C) followed by the 24 h incubation at 4°C. Leaf tissues were collected at 0, 2, 4, 6, 8, 10, and 24 h time points during the interval of each treatment. Leaves, including petioles, were collected from the medium third portion of the shoot. Stem xylem was isolated from young poplar shoots by making longitudinal cuts and removing surrounding layers of epidermis, bark, cortex, phloem, and cambium. Roots were extensively washed to remove soil and briefly dried using filter paper before freezing in liquid nitrogen. All tissues (with the exception of short term heat stress) were collected at the same time of day 11 am and immediately frozen in liquid nitrogen.

### RNA Extraction, Preparation and Sequencing of RNA-Seq Libraries

Total RNA was extracted from each replicate as previously described (Filichkin et al., 2010). 81 individual strand-specific RNA-Seq libraries were prepared using True-Seq kit according to the manufacture’s protocols (Illumina, Inc., San Diego, CA, United States). Sequencing was performed at the core facilities of Oregon State University Center for Genome Research and Biocomputing^1^ using paired-end 101cycles runs and Illumina HiSeq 2000 platform.

### Initial Analysis of RNA-Seq Datasets

*Populus trichocarpa* genome assembly (Tuskan et al., 2006) and gene annotations were downloaded from the Phytozome (Goodstein et al., 2012) Version 3.0 database^2^. A total of approximately 10^9^ of 101 nt paired-end RNA-Seq reads were aligned against the Version 3.0 poplar genome using STAR (Spliced Transcripts Alignment to a Reference) (Dobin et al., 2013). Only uniquely aligned reads were used in downstream analyses. For the basic expression analysis, RNA-Seq reads were aligned to the poplar V 3.0 genome using TopHat version 2.1.1 with default options. Transcripts were assembled using Cufflinks version 2.2.1 with default parameters as described^3^. The Cufflinks-generated output files in GTF format were loaded for display in Poplar Interactome project web site^4^ using the GMOD GBrowse software Version 2.55. Raw RNA-Seq data sets deposited at EMBL-EBI ArrayExpress (accession number E-MTAB-5540). Additional RNA-Seq-associated data available to public through GBrowse at Poplar Interactome project web site^4^.

### Identification of Differentially Retained Introns

Differential intron retention events (DIRs) were detected and quantified using the iDiffIR software package^5^ (Xing et al., 2015). A cutoff of <0.05 for adjusted *p*-values was used to filter statistically significant DIR events. The adjusted log-fold change of intron coverage was calculated as the log fold change adjusted to a pseudo-count divided by a measure of standard error as described^5^. The best pseudo-count value was calculated using the pseudo-count for which the adjusted log-fold change was minimized. To minimize effects of fluctuating levels of transcript expression on DIR calls only transcripts showing fivefold or less expression change across the treatments were used for DIR value calculation. The relative IR scores were calculated as the average read depth of an intron divided by the number of splice junction reads that flank the intron. The splicing ratio difference for DIRs was calculated as the difference between the ratio scores in the control and the treatment.

### Quantification of DIR-Harboring and Fully Spliced Transcript Isoforms

Reverse transcription followed by droplet digital PCR (RT-ddPCR) was performed using Bio-Rad (Bio-Rad, Hercules, CA, United States) iScript Select cDNA synthesis and QX200 EvaGreen ddPCR kits, respectively, according to the manufacturer protocols^6^. SJ-specific primers (see Supplementary Material for primer sequences) were designed using previously described strategy (Filichkin et al., 2010, 2015a).

### Production and Analysis of Iso-Seq Libraries

For Iso-Seq libraries equal amounts of total RNA from biological replicates which were used for RNA-Seq libraries were combined to generate six RNA pools: leaf control, leaf stress, root control, root stress, xylem control, xylem stress. Each stress pool included all replicates of stress treatments (e.g., heat, cold, drought, and salt). Complementary DNA (cDNA) was synthesized and Iso-Seq libraries generated according to standard procedures^7^. cDNA from each sample was divided into four fractions (approximately in 1–2, 2–3, 3–6, and 5–10 Kbp ranges) and sequenced using Pacific Biosciences RSII system and Single Molecule, Real-Time (SMRT) Sequencing SMRT cells essentially as described^7^. 1–2 Kbp libraries were run using two SMRT cells whereas libraries from larger fractions were run once. Initial processing of the raw Iso-Seq sequence data assembly was performed at Arizona Genomics Institute essentially as described^8^. Primary Iso-Seq data analysis was performed using the SMRT pipeline (Version 1.87.139483). Initial alignments of Iso-Seq reads to poplar genome for plotting on GMOD GBrowse were produced using STAR aligner V.2.5.2a (Dobin et al., 2013). STAR output files in BAM format were uploaded using GMOD GBrowse tool (V.2.55).

Final analysis of Iso-Seq data was performed as follows. Reads of insert predicted as non-full length by the SMRT Analysis software from Pacific Biosciences that did not exhibit an identifiable poly-(A) tail and 3′ adapter were removed. Finally, Iso-Seq reads were corrected using a hybrid error correction method LoRDEC (Salmela and Rivals, 2014) against a de Bruijn graph constructed from the bulk of RNA-Seq libraries (approximately 10^9^ of 101 nt paired-end RNA-Seq reads, see above). These corrected Iso-Seq reads were further aligned to the poplar genome using Transcriptome Analysis Pipeline for Isoform Sequencing (TAPIS) (Abdel-Ghany et al., 2016) pipeline to produce approximately 10^6^ aligned reads. Iso-Seq alignments were then assembled into strand-specific clusters and unique splice isoforms were inferred by merging alignments with common splice junctions.

For identification of IR events in miRNA precursors all Iso-Seq read clusters (15,087) were first aligned to the poplar genome assembly V.3.0. Iso-Seq Read clusters that did not overlap any annotated genes were aligned using BLAST search against plant hairpin miRNA from miRBase^9^. Total of 335 of these read clusters showed a significant match (at *e*-value < 10e^-5^). 192 out of the 335clusters showed a significant match against annotated *P. trichocarpa* miRNAs whereas 123 matched annotated *P. euphratica* miRNAs. Iso-Seq read alignments to the poplar genome and transcript isoform models publicly available through Poplar Interactome project web site^10^.

## Results

### Strategy and Computational Approaches Used for Profiling of Stress-Induced Splice Isoforms

To ensure comparable representation of both short- and long-term phases of stress response and provide statistical means of data analysis our experimental design included following key arrangements. 81 RNA-Seq libraries representing triplicates of short term and prolonged phase of each stress treatment for leaf, root, and stem xylem were generated as described in Section “Materials and Methods” (**Supplementary Files 1**, **2**). Both RNA-Seq and Iso-Seq libraries were produced using the same RNA samples. Stress-inducible DIR events and splice junctions were mapped using RNA-Seq datasets whereas Iso-Seq was used for general survey and/or validation of splice isoforms structure predicted by RNA-Seq. Both data sets were used to build independent transcript isoform models (see section “Materials and Methods” and **Supplementary File 1**). Likewise, for quantifying absolute copy numbers of isoforms by Droplet Digital PCR, we employed same RNA samples used for libraries production. A single source of RNA input in all experiments ensured a consistent approach for downstream validation and statistical analyses.

### Intron Retention Is a Dominant Class of Alternative Splicing Events across All Tissue Types

To evaluate the distribution of the major classes of AS events we analyzed individual cDNA isoforms from untreated controls or stress-treated tissue samples using Iso-Seq data. The RNA from control or stress-treated tissues types were pooled to produce six Iso-Seq libraries as described in Section “Materials and Methods.” First, non-full length reads that did not exhibit an identifiable poly-A tail were removed. Finally, corrected full-length non-chimeric Iso-Seq reads were aligned to the poplar genome to produce approximately 10^6^ alignments nearly evenly distributed across tissue types and stress treatments (**Supplementary File 3**). Analysis of these Iso-Seq reads aligned to the annotated poplar genes using TAPIS software (Abdel-Ghany et al., 2016) identified 20,504 isoforms with IR, 12,658 with alternative acceptor (Alt 3′), 9,378 with alternative donor (Alt 5′), and 3,288 with ES (including mutually exclusive exons) events. Among all detected alternatively spliced transcripts intron retaining isoforms occurred with the highest frequency (44.7%) followed by alternative acceptor (27.6%), the alternative donor (20.5%) and ES (7.2%) (**Figure 1A**). Thus, similar to other plants [e.g., *Arabidopsis*, (Filichkin et al., 2010, 2015b)] the IR and ES events in *Populus* species represent the most and the least prevalent classes of AS, respectively. Additional analysis of Iso-Seq reads that aligned to non-annotated genome portions resulted in the discovery of 15,087 novel transcribed regions of which 164 were alternatively spliced (**Supplementary File 4**).

### Mapping of Novel Splice Junctions Using High Depth RNA-Seq Coverage

Paired-end Illumina libraries were analyzed for differential splicing events using the following strategy. First, reads mapping to the multiple loci in the genome assembly were removed. Second, potential false-positive splice junctions were filtered using classifier module of SpliceGrapher package (Rogers et al., 2012). Third, putative novel splicing events were predicted using SpliceGrapher software (Rogers et al., 2012). Finally, DIR events were identified using the iDiffIR software (Xing et al., 2015) (also see section “Materials and Methods”). Alignment of 101 nucleotides (nt) paired end reads to the *P. trichocarpa* genome V. 3.0 produced a total of approximately 1.1 billion uniquely aligned reads from 81 libraries from three tissues namely leaf, xylem, and root, each subjected to four stress treatments. Uniquely mapped reads comprised on average 89% per library with a typical mapped length of 196 nucleotides (**Supplementary File 5**). Using the STAR read-mapping software (Dobin et al., 2013) we detected a total of 526,014,279 transcript splice junctions (SJs) of which 15,244,125 were novel when compared with the reference *P. trichocarpa* genome V3.0 annotations. Canonical GT/AG dinucleotide splicing signals constituted a major fraction of the 516,154,103 introns whereas GC/AG and AT/AC signals represented minor proportions with 7,313,526 and 346,044 SJs, respectively.

### Abiotic Stresses Trigger Broad Spectrum of Unique and Distinct DIR Events

A total of 4,287 iDiffIR-predicted differentially retained introns showed statistically significant (*P*_adj_ < 0.05) stress-induced IR across all the treatments and tissue types (**Table 1** and **Supplementary Files 6**, **7**). A typical distribution of multivariate analysis data is shown in **Supplementary File 8**. A total of 1,654 unique genes (*P*_adj_ < 0.05) were associated with stress-induced DIR events (**Figure 1B** and **Supplementary Files 9**, **10**) with an average of 1.1 DIRs per gene. Of these, 1021 were induced by cold, 990 by drought, 942 by high salinity, and 651 by heat stress including both short- and prolonged treatment durations combined across leaf, root, and xylem tissues (**Supplementary File 11**). Of these gene sets the largest number of unique DIRs was observed for drought stress with a total of 312 genes, followed by 290 genes for cold, 243 genes for heat, and 181 genes for high salinity stress treatments. Fifty-three DIR-associated genes were observed across all stress treatments and tissue types (**Figure 1B** and **Supplementary File 11**). Drought, high salinity, and cold treatments universally affected 160 common DIR-associated genes. High salinity, heat, and cold treatments shared the lowest proportion of common DIR- associated genes (**Figure 1B** and **Supplementary File 12**). Short- and long-term phases of treatment within the same tissue type induced both unique and common subsets of DIRs (**Figure 1C** and **Supplementary File 13**). Several instances showed that the same gene could be associated with multiple DIR events that are regulated by the specific stress treatments in an independent manner.

**Table 1:** Distribution of stress-induced differential intron retention events in poplar tissues.

Tissue	Treatment		Differential intron retention events		
			
			Unique events	Associated non-redundant loci	Loci associated with multiple DIRs (two or more events per gene)
Leaf	Drought	Short term	195	174	19
		Prolonged	178	170	6
	Salt	Short term	134	112	15
		Prolonged	117	104	11
	Heat	Short term	116	103	11
		Prolonged	110	101	7
	Cold	Short term	117	110	4
		Prolonged	140	130	91

Root	Drought	Short term	172	171	1
		Prolonged	137	123	11
	Salt	Short term	166	165	1
		Prolonged	161	161	9
	Heat	Short term	150	141	8
		Prolonged	167	156	8
	Cold	Short term	213	208	5
		Prolonged	154	152	2

Xylem	Drought	Short term	203	173	20
		Prolonged	218	179	28
	Salt	Short term	249	228	18
		Prolonged	221	172	33
	Heat	Short term	222	196	19
		Prolonged	190	158	23
	Cold	Short term	244	227	15
		Prolonged	313	294	16


### Switch between Increased or Decreased Intron Retention Specifically Regulated by Stress Type

**FIGURE 2:**
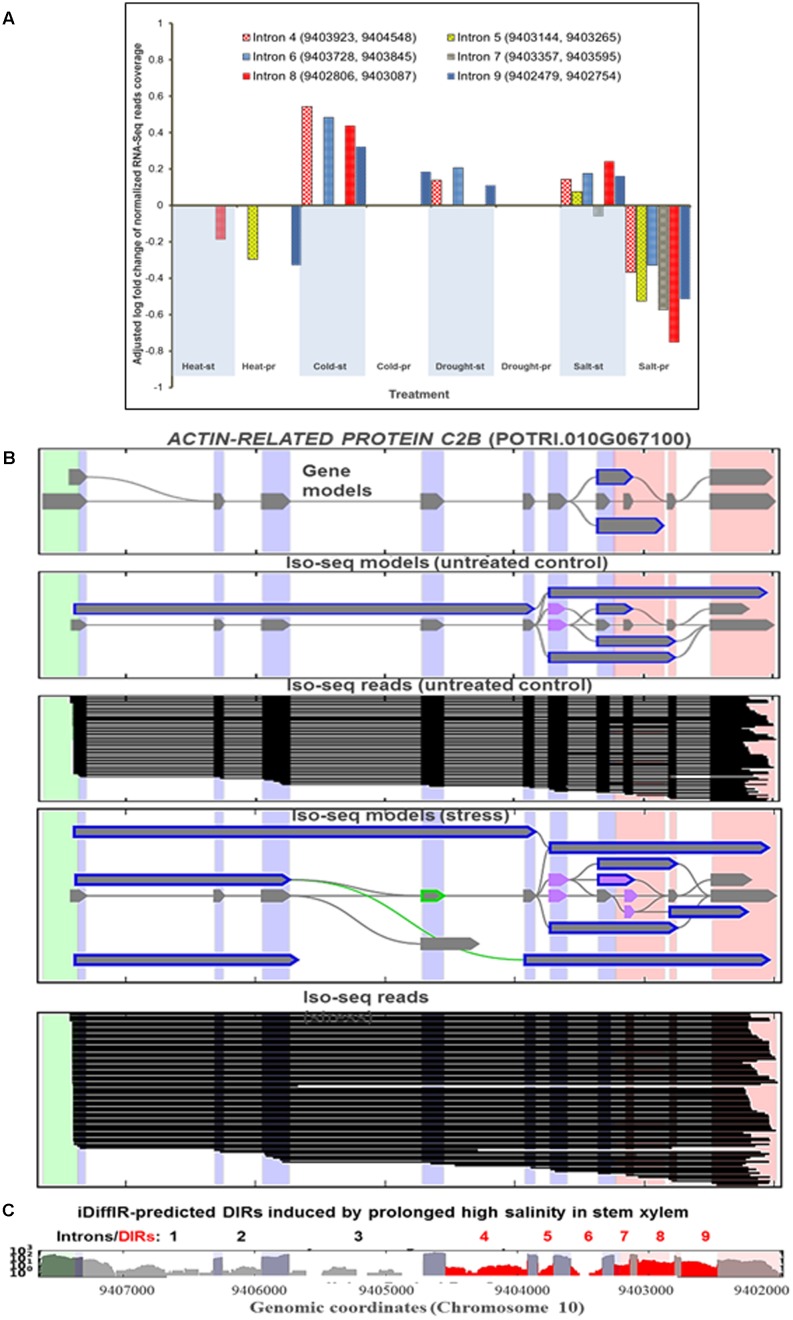
Multiple differential intron retention events can be associated with a single gene and conditionally and differentially regulated within the same tissue type in a stress-specific manner. **(A)** Change in normalized RNA-Seq coverage of DIRs harbored by transcripts derived from *ACTIN-RELATED PROTEIN C2B* gene (*ptAC2B*, *POTRI.010G067100)* in stem xylem. Six out of nine introns DIRs show statistically significant (*P*_adj_ < 0.05) increase or decrease of coverage by normalized RNA-Seq reads in a stress type-dependent manner. Vertical axis shows an adjusted log fold change of normalized RNA-Seq reads coverage of differentially retained introns. Note that five out of six DIRs (except intron 7) can be up- or down-regulated depending on type of stress treatment. **(B)** Iso-Seq splicing models (ribbon drawings) and individual single molecule reads (black lines) of *ptAC2B* mRNA. Iso-Seq models suggest an extensive diversification of splice isoforms under abiotic stress. **(C)** Normalized RNA-Seq coverage of DIR events (shown in red) in *ptAC2B* mRNA. DIR events predicted from the RNA-Seq data using iDiffIR consistently corroborated by Iso-Seq models and individual reads. *ptAC2B* DIR events detected only in xylem but not in other tested tissue types. The vertical axis in denotes log of normalized RNA-Seq reads coverage.

We further examined if a switch between increasing or decreasing IR levels specifically pre-determined by stress type. Using the iDiffIR software developed for detecting differentially retained introns, we identified a set of 290 genes associated with two or more DIRs induced by broad range of treatments (**Supplementary Files 14**, **15**). On average, each of these genes was associated with ∼2.4 differentially regulated introns. We further examined instances of stress- and/or tissue-dependent regulation of such multiple DIR-harboring mRNAs. For example, an increase or a decrease of IR events in mRNA of *actin-related protein c2b* (*ptAC2B*) gene (*POTRI.010G067100*) in xylem was specifically determined by the type of treatment (**Figure 2A**). The short phase of high salinity stress resulted in an increase of the IR in five out of six DIR events harbored by *ptAC2B* mRNA whereas the prolonged phase caused a substantial decrease in all six events. In contrast, retention of intron seven decreased during the short phase of the treatment suggesting that short- or long-term treatment phases can have an opposite effect on regulation of distinct DIR events in the same mRNA. The structure of intron-retaining transcript models was generally corroborated by both RNA-Seq and Iso-Seq data (see **Figures 2B,C** for *ptAC2B* transcript models and for additional examples in Supplementary Material) providing an additional cross-platform validation of our computational methods. Similar to *ptAC2B*, the retention of six out of ten predicted introns of putative mRNA encoding *glutamate ammonia ligase* (*ptGAL*, *POTRI.004G085400*) in leaf tissues occurred in a differential and stress-specific manner (**Supplementary File 14**). Similar stress type-dependent behavior of DIRs harbored by *ptGAL* mRNA also occurred in xylem tissue (data not shown). We found numerous additional instances where the outcome of a particular IR event specifically regulated by stress type (**Supplementary File 14**).

### Retention of Individual Introns in mRNA with Super Stress-Responsive DIRs Can Be Regulated Independently in a Stress-and/or Tissue Specific Manner

Among transcripts harboring multiple DIR events, several mRNAs displayed a broad stress response across all stress treatments (i.e., at least one DIR event was detected during one or more treatments). Such transcripts with highly responsive DIRs across all stress treatments were associated with 21, 6, and 33 genes in leaf, root, and xylem tissues, respectively (**Supplementary File 16**). Three genes out of 179 multi-DIR-associated loci responded universally to all tested stress treatment through the retention of one or more introns. Such super stress-responsive genes included orthologs of *Arabidopsis DCD (*DEVELOPMENT AND CELL DEATH*)* (*POTRI.003G141900*), a *HEAT SHOCK TRANSCRIPTION FACTOR B1* (*POTRI.007G043800*), and a putative *PATATIN-RELATED PHOSPHOLIPASE* (*POTRI.007G040400*). All three super stress-responsive poplar genes are likely to be involved in regulation of stress responses because their *Arabidopsis* orthologs and homologs were also implicated in cellular responses to a broad range of environmental stresses (Huang et al., 2008; Li et al., 2011; Kim et al., 2013). Another *dcd*-related gene, *ptDCD-l* (*POTRI.001G088800*), was among other five genes commonly responsive to drought, cold, and salt (**Supplementary File 16**). Three out of four introns in *ptDCD-l* mRNA were retained in xylem in various arrangements depending on type of stress treatment. Combinatory arrangements and the degree of retention of multiple DIRs associated with transcripts encoding *GLUTAMATE AMMONIA LIGASE* (*POTRI.004G08540*0) in leaf tissues were also specifically controlled by the type of treatment (**Supplementary File 14**). Similar to *ptAC2B*, particular DIRs in the *ptDCD-l* mRNA were regulated by several stresses independently of each other (**Supplementary File 14**). *ptAC2B* and *ptGAL* transcripts harbored stress-inducible DIRs only in xylem and leaf, respectively, but not in any other tissues types. These results suggest that up- or downregulation of each event in multi-DIR transcripts in principle can occur independently in a stress- and/or tissue-specific manner.

### mRNAs Encoding Key Regulatory Proteins Often Harbor Stress-Induced DIR Events

Numerous stress-induced differentially retained introns were present in the transcripts of key gene families regulating pre-mRNA splicing, general and specific stress-responses, plant development, cell wall metabolism, and circadian rhythms. Of the 101 annotated poplar splicing factors approximately 13% contained DIRs. DIR events in poplar mRNAs encoding orthologs of human general AS regulator SF2/ASF (*ptSF2*) and mammalian splicing factors 9G8 (*ptSRZ22*) harbored complex combinations of DIRs and other AS events in the 3′ portions of the transcripts. DIRs in *ptSF2* and *ptSRZ22* mRNAs were specifically induced by the heat stress and detected only in xylem tissues (**Supplementary File 9**).

At least one DIR per major gene family was found in general regulators of stress-response such as ABA responsive element binding (*AREB*), dehydration responsive element binding (*DREB*) TFs, and 14-3-3 genes (**Supplementary File 10**). DIR events in poplar genes encoding *ptAREB* and *ptDREB* factors were induced primarily by thermal and/or drought stress. Transcripts of numerous heat-shock factors also contained temperature-, drought-, and salt-sensitive DIRs (**Supplementary File 17**). Among key genes controlling plant development, poplar ortholog of *Arabidopsis late embryogenesis abundant 2* displayed a particularly interesting pattern of stress-regulated IR. Retention of a single intron in *ptLEA* transcript was up or down regulated in a stress- and/or tissue-specific manner (**Supplementary File 18**).

Numerous genes regulating cell wall metabolism including cellulose and lignin biosynthesis also harbored DIR events. Among genes involved in cellulose and lignin biosynthesis pathways, we identified seven and three transcripts harboring DIRs, respectively. For instance, an inclusion of the first intron in mRNA encoding GLYCOSYL TRANSFERASE FAMILY 8 PROTEIN (*POTRI.005G218900*, a co-ortholog of *Arabidopsis At1g06780* and *At2g30575* genes) was up-regulated by the prolonged heat treatment (**Supplementary File 19**). Such up-regulation occurred only in stem xylem but not in other tested tissues (data not shown) suggesting that this DIR event is xylem specific. DIR events in the transcripts of several key circadian regulators were regulated by several stress treatments (**Supplementary File 20**). At least 17% of annotated poplar genes from *glycine rich protein-like* family were associated with one or more DIRs.

### Stress-Coordinated Modulation of Intron Splicing Ratios in Co-regulated DIR Clusters

**FIGURE 3:**
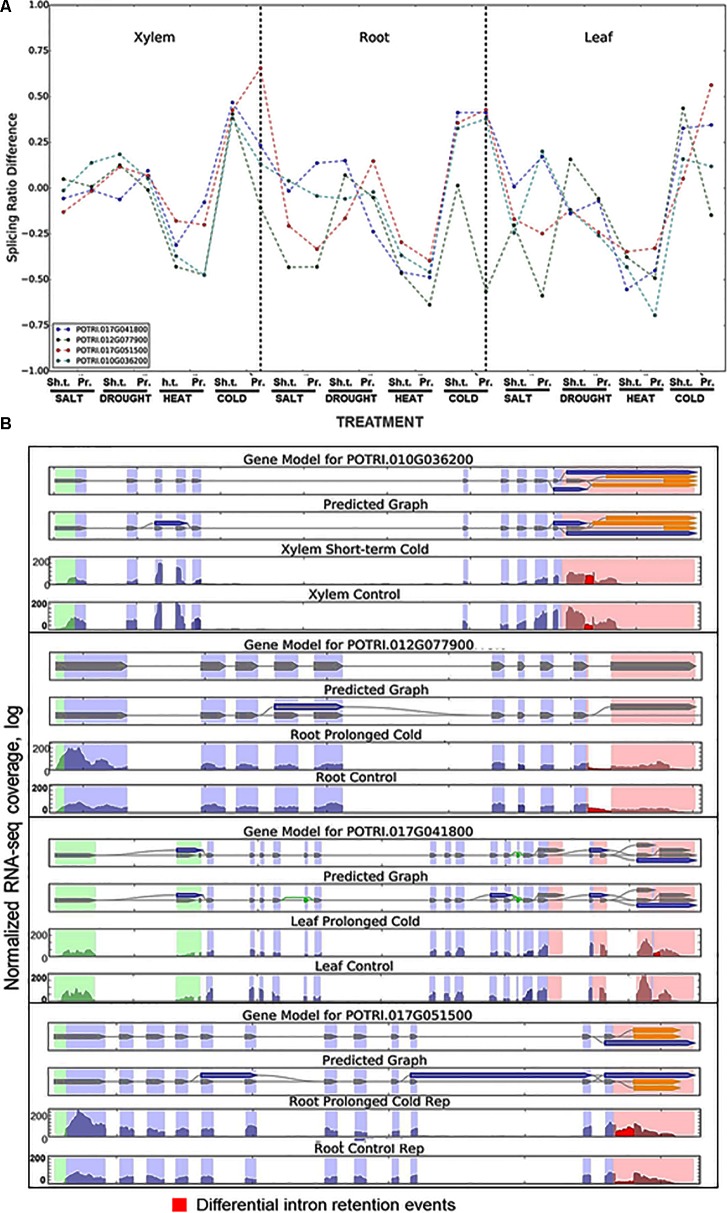
A representative example of DIR events regulated across all the treatments in all tissue types in a coordinated manner. **(A)** Intron splicing ratios show similar profiles across all treatments and tissue types. Differences in intron splicing ratios were calculated as described in Section “Materials and Methods.” The cluster includes a *40 KDA HEAT SHOCH PROTEIN* (*POTRI.010G036200*), *SERINE PROTEASE* (*POTRI.012G077900*), *DYNAMIN* (*POTRI.017G041800*), and *SAICAR SYNTHASE* (*POTRI.017G051500*). **(B)** Predicted models and DIR events of the genes in the cluster shown in **(A)**. Note that all DIRs confined to the transcript 3′ end. Retention of these introns will generate transcripts with unusually long (>300 nt) 3′UTRs – potential targets for NMD degradation. Transcript models and DIR events were predicted using SpliceGrapher and iDiffIR software packages, respectively. RNA-Seq read coverage of DIRs depicted in red. Sh.t., short term treatment; Pr., prolonged treatment.

To characterize changes in IR rates across the transcriptome we calculated the ratios of intron inclusion in stress treated tissues vs. untreated control samples. The relative IR rates were calculated using the RNA-Seq read coverage of the introns and corresponding splice junctions. The score of relative IR rate was calculated as the average of intron read coverage divided by the number of splice junction reads that are associated with this particular intron. The difference in splicing ratios was calculated as the difference between the ratio scores in the control and the treatment as described in Section “Materials and Methods.” Intron splicing ratios of DIRs across all stress treatments for leaf, root, and xylem tissues summarized in **Supplementary Files 20**–**22**, respectively. Clustering of differential splicing ratios produced 124 DIR events co-regulated across the stress treatments (**Supplementary Files 23**–**25**). **Figure 3** shows a typical example of a cluster of co-regulated DIR events. The cluster includes mRNAs encoding 40 KDA HEAT SHOCK PROTEIN (*POTRI*.010G036200), SERINE PROTEASE (*POTRI.012G077900*), DYNAMIN (*POTRI*.017G041800) and SAICAR SYNTHASE (*POTRI*.017G051500). A distinct feature of this cluster is that all DIRs localized to the 3′ ends of mRNAs (**Supplementary File 21**). Retention of these introns will result in mRNAs with unusually long 3′ untranslated regions (3′UTR) — potential targets for NMD degradation (Kertész et al., 2006). Splicing ratios of each DIR showed either increase (e.g., a higher proportion of intron-retaining transcript) or decrease (e.g., a lower proportion of transcript with retained intron) across all stress types and tissues (**Figure 3**). Profile similarities for this cluster were especially obvious during short term and prolonged phases of heat (ratio decrease) or cold (ratio increase) stress. Statistically significant (*P* < 0.05) coordinated changes in intron splicing ratios were observed in other 124 independent gene clusters (**Supplementary File 26**) suggesting that many DIRs are co-regulated in a stress-specific manner.

### Dynamics of DIR-Harboring Isoforms Accumulation

**FIGURE 4:**
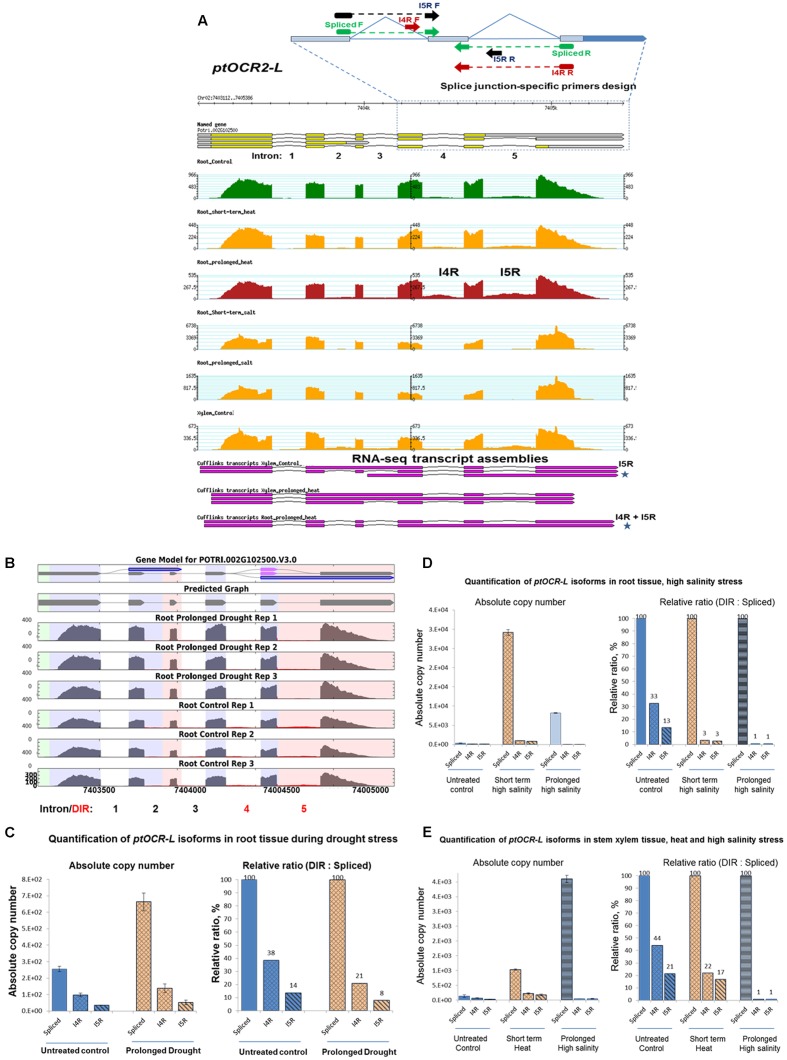
Dynamics of stress-driven IR in mRNA encoding poplar ovarian cancer related transcription factor (*ptOCR2-L*, *POTRI.002G102500*). This example illustrates that stress-induced increase in copy number of the fully spliced *ptocr-lptocr-lptocr2-l* mRNA occurs concomitantly with a decrease in the relative ratios of both retained introns. **(A)** RNA-Seq coverage of *ptocr-lptocr-lptocr2-l* locus and design of splice junction (SJ)-specific primers. Primers designated as “Spliced” detect only the isoform with fully spliced introns 4 and 5 (gene model *POTRI.002G102500.1*); “I4R” – intron 4 is retained whereas intron 5 is spliced (reverse primer spans I4 SJ); “IR5” – I5 is retained whereas I4 is spliced (corresponds to gene model^.^3; forward primer spans I4 SJ). Stars indicate Cufflinks transcript assemblies supporting retention of both fourth and fifth introns (I4R + I5R) under the heat stress in roots and the fifth intron (I5R) in xylem. *Y*-axis indicates the number of Illumina reads aligned to the genome. **(B)** Gene model and differentially retained introns of *ptOCR2-L* transcripts. **(C–E)** Quantification of mRNA absolute copy number and relative ratios of the differentially retained introns vs. fully spliced transcripts in tissues under various stress treatments. Absolute copy number of mRNA was quantified using reverse transcription – droplet digital PCR (RT-ddPCR) and event-specific primers as described in Section “Materials and Methods.”

To examine stress-induced changes in quantities of transcript splicing isoforms, we further analyzed IR rates using real-time quantification of individual isoforms copy number. To achieve this, we employed reverse transcription – droplet digital PCR (RT-ddPCR) using event-specific primers as described in Section “Materials and Methods.” RT-ddPCR allows quantification of relative proportions of splice variants in the same cDNA sample without the need for a standard curve (Baker, 2012). Using examples of two DIR-harboring mRNAs encoding conserved metazoan TFs we investigated stress-driven shifts in ratios between their fully spliced and intron-retaining isoforms. *ptOCR2-L* (*POTRI.002G102500*) mRNA encodes an ortholog of *Arabidopsis ONCOGENE-RELATED PROTEIN 2*, (*AT4G24380*) whereas *ptTFIIB* mRNA (*POTRI.006G048400*) encodes an ortholog of the conserved eukaryotic *TRANSCIPTION INITIATION FACTOR II SUBUNIT B*. Both *OCR2* and *TFIIB* proteins are involved in regulation of eukaryotic cellular responses to stress (Gong et al., 2001) (Zanton and Pugh, 2006). DIR events in both *ptOCR2-L* and *ptTFIIB* transcripts showed fluctuations in the degree of IR in a stress-specific manner (**Supplementary File 22**). A sharp increase in the levels of *ptOCR2-L* mRNA occurred under specific stress treatments and only in particular tissue types (**Figure 4A**). The inclusion of both or any of the fourth (I4R) and/or fifth (I5R) introns would produce either PTC+ mRNAs or transcripts with abnormally long 3′UTRs which could be potential NMD targets. To examine stress-induced shifts in ratios between intron-retaining and fully spliced transcripts we quantified the accumulation of the I4R and I5R isoforms under drought, high salinity or heat stresses in roots and xylem tissues. The exact quantification of *ptOCR2-L* isoforms using reverse transcription – droplet digital PCR (RT-ddPCR) and event-specific primers revealed that high salinity treatment induced a sharp increase in absolute copy numbers of the fully spliced mRNA in root and xylem. Such an increase was accompanied by a moderate increase in I4R and I5R isoforms copy number (**Figures 4B,C**). However, a sharp increase in spliced transcript copy number was also accompanied by a decrease in the relative abundance of intron-retaining isoforms, calculated as a percentage of absolute copy number of intron-retaining isoform relative to the fully spliced mRNA. Similarly, the copy number of the fully spliced *ptOCR2-L* mRNA and its intron-retaining isoforms were consistently increased in root and xylem tissues under short term heat and prolonged drought stresses (**Figures 4C–E**). In contrast, the relative ratios between both intron-retaining isoforms and spliced mRNA sharply decreased under heat and drought treatments. Thus, for all three types of treatment (e.g., high salt, heat, and drought) a sharp increase in absolute copy numbers of fully spliced *ptOCR2-L* mRNA followed by a decrease in relative ratios of intron-retaining isoforms.

**FIGURE 5:**
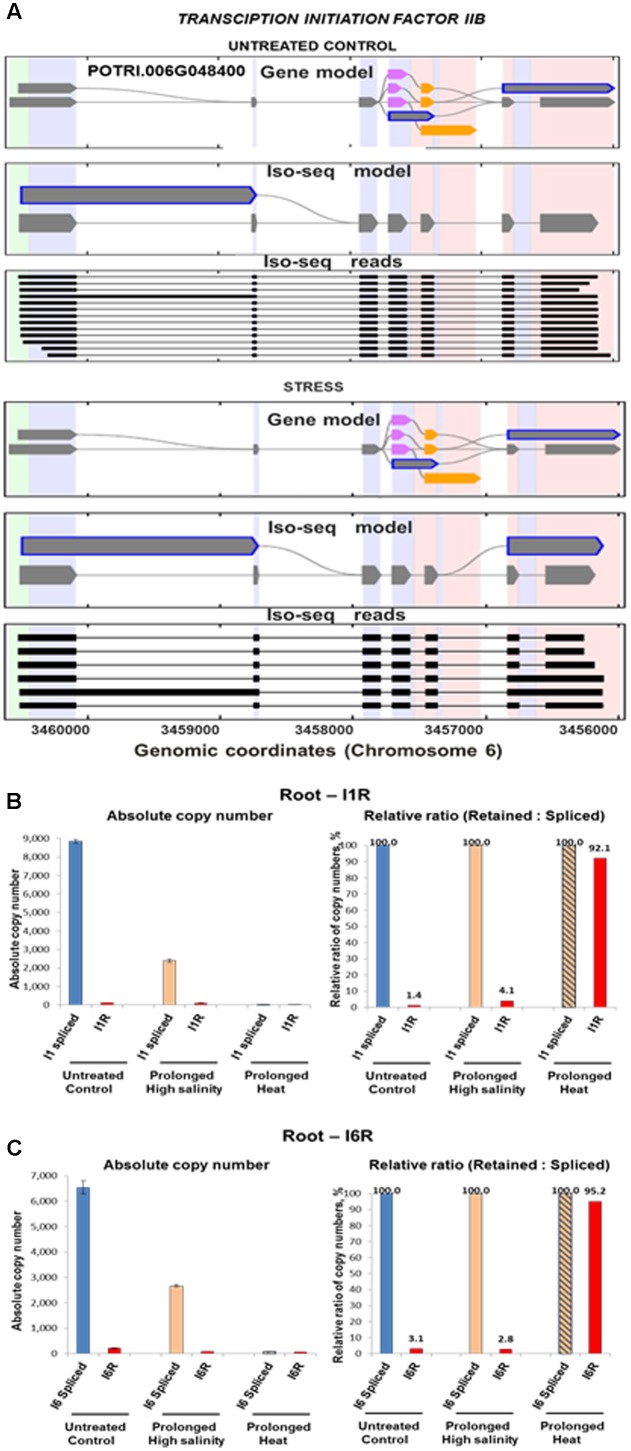
Stress-driven increase of fully spliced *pttfIIB* mRNA accompanied by the switch in relative ratios of intron-retaining isoform. Quantification of absolute copy number of *ptTFIIB* transcript isoforms encoding *P. trichocarpa* general TRANSCIPTION INITIATION FACTOR IIB (*POTRI*.006G048400). **(A)** Iso-Seq isoforms models of mRNA under normal conditions and under abiotic stress (combined treatments). **(B,C)** The degree of retention of the first and sixth introns of the *pttfIIB* mRNA regulated by the prolonged salinity and heat in a stress-specific manner. Both the first and the sixth introns can be partially or completely retained producing isoforms with early PTCs or with unusually long 3′ untranslated regions, respectively. Notably, absolute copy number of isoforms retaining either first (I1R) or sixth (I6R) introns decreased in parallel with the salt- and heat-induced down-regulation of the fully spliced mRNA. However, a decrease of copy number of the fully spliced *pttfIIB* mRNA was accompanied by the increase of relative proportions of both (e.g., I1R and I6R) intron-retaining isoforms. Thus, prolonged heat stress drastically increased the relative ratios of I1R and I6R isoforms to the spliced mRNA despite increase of copy number across all isoforms. Absolute copy number was quantified using reverse transcription – droplet digital PCR and event-specific primers as described in Section “Materials and Methods.”

We further examined the stress-driven regulation of isoforms ratios of *ptTFIIB* (POTRI.002G102500), a poplar ortholog of mammalian general TF *TFIIB*. The *ptTFIIB* transcripts showed tissue-specific and/or stress-dependent retention of its first (I1R) and/or the sixth (I6R) introns. Iso-Seq models strongly support such stress-modulated retention of these two introns (**Figure 5A**) (**Supplementary File 22**). Prolonged high salinity and heat treatments sharply increased levels of *ptTFIIB* mRNA in roots. Quantification of individual isoforms using RT-ddPCR showed that the retention of both introns decreased together with stress-induced down-regulation of the spliced mRNA. Conversely, a decrease in the copy number of fully spliced mRNA under heat stress was associated with a sharp increase of relative retention rates for both introns (92 and 95% for the I1R and the I6R events, respectively, see **Figures 5B,C**).

Stress-induced fluctuation of *ptTFIIB* mRNA of such extreme amplitude was observed only in root tissues under specific stress conditions such as prolonged high salinity and heat stresses (**Figures 5B,C**). In contrast to roots, the *ptTFIIB* mRNA levels in leaves showed more moderate changes under temperature stress. We used the leaf tissue to evaluate dynamics of DIR fluctuations in *ptTFIIB* isoforms in more narrow range using high resolution heat-cold stress time course (**Supplementary File 27**). To mimic extreme temperature swings, plants were subjected to the cyclical temperature changes as follows. First, plants were treated at 42°C for 24 h followed by the transition to 4°C for another 24 h. The absolute copy number of the first intron-retaining and fully spliced *ptTFIIB* isoforms was monitored using RT-ddPCR. During a 24-h segment of heat treatment, fully spliced mRNA remained at near constant levels. In contrast, accumulation of the I1R transcript showed a substantial copy number increase. The relative ratio of I1R isoform to the spliced mRNA also showed a considerable increase under heat stress (**Supplementary File 13**). The following 24 h cold treatment segment resulted in a decrease of both the I1R transcript and its ratios relative to the highest copy number observed during heat stress. Thus, the copy number of the fully spliced or intron-retaining isoforms increased or decreased during the heat or cold treatments, respectively. Concomitantly, during heat stress the relative ratio of intron-retaining isoform increased approximately sevenfold. Conversely, the cold treatment segment showed the decrease of both isoforms and reduction of the relative ratios to approximate levels of untreated controls. This result is consistent with a general conclusion of *ptTFIIB* and *ptOCR2-L* case studies that specific stresses induce broad fluctuations in relative ratios of the intron-harboring isoforms to the fully spliced mRNA.

### Transcripts of Many Non-protein Coding Genes Alternatively Spliced and Harbor Differentially Retained Introns

A total of 15,087 Iso-Seq read clusters with low protein coding capacity that did not align to any annotated genes in poplar genome assembly (V.3.0) were designated as non-protein coding transcripts. Sequence alignments of these reads to hairpin miRNAs from miRBase showed a significant match of 335 reads to annotated plant miRNAs (*e*-value < 10e^-5^). 192 Iso-Seq reads had a significant hit against annotated *P. trichocarpa* miRNAs whereas 123 showed a significant match to *P. euphratica* miRNAs. Mapping splice junctions revealed that many of these primary miRNA transcripts undergo extensive AS including stress-specific IR events. Examples of splicing models for ptc-miR156e and ptc-miR398c are shown in **Supplementary File 28** whereas features of other primary non-coding transcripts can be explored using Poplar Interactome GBrowse^11^.

## Discussion

### High Resolution Survey of Poplar Transcriptome Reveals Thousands of New Stress-Induced Transcript Isoforms of Protein-Coding and Non-protein Coding Genes

We carried out high depth coverage RNA-Seq survey of changes in AS patterns induced by drought, high salinity, heat and cold stress across transcriptomes of leaf, stem xylem and root tissues. Using more than one billion genome-aligned RNA-Seq reads from 81 libraries, we mapped approximately 500 million splice junctions to the *P. trichocarpa* genome (V.3.0). 15,244,125 of these were novel. Majority of the introns had canonical GT/AG splicing signals whereas GC/AG and AT/AC dinucleotide signals represented minor fractions. A parallel analysis of a single molecule real time sequencing (Iso-Seq) data produced about one million genome-aligned reads and showed that IR is a prevalent event across all tissues and treatments (an average of 44.7%). Alternative acceptor and donor events represented 27.6 and 20.5% of all detected AS events, respectively, whereas ES was the more rare class (7.2%).

In addition to protein-coding genes, we identified dozens of novel non-protein coding genes. Analysis of primary miRNA transcripts revealed that many of poplar pri-miRNAs undergo extensive AS including conditional IR. Since introns can be critical for proper biogenesis and processing of some *Arabidopsis* miRNAs (Szarzynska et al., 2009; Bielewicz et al., 2013), the further in-depth investigation is required to establish particular roles of miRNA DIRs in the regulation of stress responses in poplar. Both RNA-Seq and Iso-Seq datasets and catalogs of inferred transcript isoform models present valuable public resources for refining existing poplar genome annotation and data mining of stress-induced changes in transcriptomes of key poplar tissues. RNA-seq-derived expression data (**Supplementary File 29**) presents an additional public source of data mining to establish possible correlations between mRNA expression and occurrence of particular splicing events.

### Stress-Induced Differential Intron Retention Is a Common Phenomenon in Plants

In-depth analysis of the IR events showed that the degree of retention for many introns under abiotic stress is variable, an incident described here as DIR. Comparative analysis of the degree of stress-induced IR events revealed that DIR is widespread across major tissue types of Western poplar. A broad range of intersecting or stress-specific DIR events was triggered by drought, high salinity, heat or cold stress across the leaf, stem xylem and root tissues. Short-term and prolonged treatment produced subsets of intersecting (i.e., common for both phases) or phase-specific DIR events. We identified several subsets of DIRs commonly induced by two or more stress types. The highest number of genes associated with unique DIRs was induced by drought (312) followed by cold (290), heat (243), and high salinity (181). The repertoire of differentially retained introns associated with the same gene was often tissue-specific and varied across the leaf, root, and xylem tissues (**Figure 2**). Detection of transcripts with multiple but independently differentially regulated DIRs suggested that increase or decrease in the degree of IR can be specifically regulated by the type of stress. DIRs were found across main gene families including those involved in regulation of plant stress-responses, development, pre-mRNA splicing, transcription, cell wall metabolism, and circadian rhythms.

Interestingly, transcripts of many key genes involved in regulation of general and specific responses to environmental stresses harbored DIR events. Discovery of numerous stress-inducible DIRs in transcripts encoding poplar splicing factors was consistent with findings that pre-mRNAs of several *Arabidopsis* SR splicing factors undergo extensive AS (Palusa et al., 2007; Filichkin et al., 2010; Palusa and Reddy, 2010; Reddy and Shad Ali, 2011; Thomas et al., 2012; Reddy et al., 2013; Ding et al., 2014). Key regulators of plant stress responses such as TFs encoded by *AREB*, *DREB*, *NAC*, and *14-3-3* gene families alternatively spliced under stress (Nakashima et al., 2009; Lata and Prasad, 2011; Todaka et al., 2012; Zhao et al., 2014; Tian et al., 2015). Our finding that these gene families in poplar are also associated with numerous stress-induced DIR events suggests a potential role that DIR play in regulating stress responses.

### Stress-Coordinated Fluctuations of Intron Splicing Ratios Occur at Transcriptome-Wide Scale

We detected statistically significant coordinated fluctuations in intron splicing ratios across 124 independent gene clusters suggesting that many DIRs are co-regulated in a stress-specific manner. Observation of such coordinated stress- and/or tissue-dependent co-regulation across numerous DIRs suggested a broader role of DIR in transcriptome adjustments during adaptation to stresses. We quantified the exact stress-induced changes in IR rates using case studies of two key highly conserved eukaryotic TFs.

### Dynamics of Differential Intron Retention in Case Studies Suggest That Shifts in Isoform Ratios Regulated in a Stress-Specific Manner

Quantification of absolute copy number of *ptTFIIB* and *ptOCR2-L* mRNAs revealed a consistent trend in the stress-induced switching of the relative ratios between fully spliced and intron-retaining isoform. Sharp up-regulation of the fully spliced *ptOCR2-L* mRNA occurred in parallel with a decrease in the relative ratio of intron-retaining isoforms under stress. Conversely, lower absolute copy numbers of fully spliced *ptOCR2-L* mRNA in untreated controls paralleled increase of relative ratios of intron-retaining isoforms (**Figures 4C–E**). In contrast to *ptOCR2-L*, prolonged heat and salt treatments resulted in the severe decrease in the copy number of a fully spliced *ptTFIIB* mRNA. Such an extreme decrease of the fully spliced mRNA levels was accompanied by the reduction in relative proportions of intron-retaining *ptTFIIB* isoforms (**Figures 4B,C**). The high resolution heat-cold stress time course of accumulation *ptTFIIB* copies in leaf under consecutive heat/cold treatment showed a similar tendency in changing isoform ratios (**Supplementary File 27**). However, more moderate mRNA fluctuations were associated with less conspicuous changes in isoforms ratios.

Dynamics of IR in these case studies suggested a common relationship between the fully spliced and DIR-harboring mRNA isoforms. Specifically, the decrease in relative ratio of intron-retaining isoform accompanied stress-induced up-regulation of the fully spliced mRNA and vice versa. Additional in-depth studies required to establish if this notion can be applicable at transcriptome-scale level.

### Transcriptome Stress Adaptation Associated with Switches in Relative Ratios of Isoforms

About 42–60% of *Arabidopsis* genes alternatively spliced with many events being condition, developmental stage, and/or tissue type-dependent (Filichkin et al., 2010; Marquez et al., 2012). AS events that generate nonsense variants with diminished or abolished protein coding capacity often degraded by NMD [reviewed in (Popp and Maquat, 2013)]. Such splicing outcomes widespread in mammalian cells and described as “unproductive AS” (Lewis et al., 2003). It was proposed that unproductive splicing in eukaryotes play a role in the regulation of transcript abundancy via coupling with NMD pathway (Lareau et al., 2007). In plants, a dominant proportion of alternatively spliced pre-mRNAs harboring PTCs is generated through IR (Filichkin et al., 2010; Filichkin and Mockler, 2012; Kalyna et al., 2012; Marquez et al., 2012) [reviewed in (Reddy and Shad Ali, 2011; Reddy et al., 2013)]. However, a majority of these IR events with some exceptions generally are not targeted by NMD for degradation (Palusa et al., 2007; Filichkin et al., 2010; Filichkin and Mockler, 2012; Kalyna et al., 2012; Marquez et al., 2012). For instance, an mRNA encoding *CCA1*, the key regulator of plant circadian oscillator, presents a particularly interesting example of DIR. Intron-retaining *CCA1* transcripts are NMD-insensitive (Filichkin and Mockler, 2012; Filichkin et al., 2015a,b). Similar to *ptTFIIB* and *ptOCR2-L*, temperature stresses shift ratio between intron-retaining and fully spliced *CCA* isoforms (Filichkin et al., 2010; Filichkin and Mockler, 2012; Filichkin et al., 2015a). These case studies and detection of numerous gene clusters with coordinated changes in intron splicing ratios suggest that stress-induced shifts of isoforms ratios widespread in plants.

Increasing evidence indicates that translation and degradation by NMD of intron-retaining mRNAs can be prevented via reversible sequestration of splicing intermediates (Boothby et al., 2013; Gohring et al., 2014; Brown et al., 2015; Filichkin et al., 2015b). Intron removal and translation of such masked mRNAs can be triggered by the specific environmental condition (Boothby et al., 2013). Increasing evidence also suggests that the dynamically changing repertoire and extent of IR events modulate mRNA abundance in the mammalian cell (Braunschweig et al., 2014). Altogether, our results broadly consistent with this notion and favor a hypothesis that conditional IR may play a role in posttranscriptional transcriptome adjustments during adaptation to environmental stresses.

## Author Contributions

SF and PJ conceived the study and the project discussed in this paper. PD and SF carried out the greenhouse experimental setup. SF carried out all the experimental validations. MH developed the iDiffIR and TAPIS software and analyzed the RNA-Seq and Iso-Seq datasets under the supervision of AB-H and AR. MH, SF, and AB-H carried out other computational analyses. CS performed the analysis of basic gene expression, isoform transcripts assembly, and maintenance of the poplar genome browser for the project. SS assisted in RNA extraction and ddPCR experiments. SF, MH, AB-H, AR, and PJ wrote the paper.

## Conflict of Interest Statement

The authors declare that the research was conducted in the absence of any commercial or financial relationships that could be construed as a potential conflict of interest.

## Footnotes

1 http://cgrb.oregonstate.edu/core/sequencing

2 https://phytozome.jgi.doe.gov/pz/portal.html

3 http://cole-trapnell-lab.github.io/cufflinks/cufflinks/

4 http://poplar.cgrb.oregonstate.edu/cgi-bin/gb2/gbrowse/poplar/

5 http://combi.cs.colostate.edu/idiffir/

6 http://www.bio-rad.com/webroot/web/pdf/lsr/literature/Bulletin_6450.pdf

7 http://www.pacb.com/products-and-services/pacbio-systems

8 http://www.genome.arizona.edu/modules/publisher/item.php?itemid=29

9 http://www.mirbase.org/

10 http://poplar.cgrb.oregonstate.edu/cgi-bin/gb2/gbrowse/poplar/

11 http://poplar.cgrb.oregonstate.edu/cgi-bin/gb2/gbrowse/poplar/

## Supplementary Material

**FILE 1 |** Sampling strategy and flow of data analysis. **(A)** Sample collection strategy and consequent steps of data analyses to detect and validate DIR events. **(B)** Flow of data analysis for detection of splice variants using SpliceGrapher package (Rogers et al., 2012). SpliceGrapher software predicts splicing isoforms using, RNA-Seq data, expressed sequence tags alignments, and information supporting gene models.

**FILE 2 |** Descriptions of samples and stress treatments.

**FILE 3 |** Distribution of genome-aligned Iso-Seq reads across libraries and tissues.

**FILE 4 |** A list of novel transcribed regions identified by the ISO-seq data.

**FILE 5 |** Distribution of genome-aligned RNA-Seq reads across libraries and tissues.

**FILE 6 |** Lists of significant (*P*_adj_ < 0.05) DIR events with adjusted log fold change in leaf, root, and xylem across all the treatments.

**FILE 7 |** Lists of all DIR-associated genes (summarized in **Table 1**). Only unique genes associated with significant DIRs (*P*_adj_ < 0.05) are listed.

**FILE 8 |** The distribution of DIR events: typical examples of multivariate analysis (MvA) plots. **(A)** Short term root heat stress. **(B)** Prolonged heat stress in root. Colored points represent IR events with statistically significant differential retention values (P*adj* equal or lesser 0.05).

**FILE 9 |** Examples of temperature-inducible DIRs in transcripts encoding ptSF2 and ptSRZ22 splicing factors. ptSF2 (*POTRI.T134200*, top panel) is an ortholog of a human general/alternative splicing factor SF2/ASF SF2 and co-ortholog of *Arabidopsis* serine/arginine-rich proteins R34/SR1 (*At1g02840*) and SR34B (encoded by *At4g024302*). ptSRZ22 (*POTRI*.*018G009100*, bottom panel) is an ortholog of a mammalian 9G8 SF and the *Arabidopsis* serine/arginine-rich protein AthRSZp22 (*AT4G31580*). *Y*-axis shows the log of normalized intron coverage by RNA-Seq reads.

**FILE 10 |** Examples of stress-inducible DIRs in mRNAs of *ABA-responsive element binding factors (AREB) and dehydration-responsive element binding (DREB)* gene families. *Y*-axis shows the log of normalized intron coverage.

**FILE 11 |** List of genes associated with multiple DIRs.

**FILE 12 |** Intersect lists of all DIR-associated genes by stress and tissue type.

**FILE 13 |** Venn diagrams representation of overlaps between the genes associated with DIR events induced by short term or prolonged treatment phases.

**FILE 14 |** Distribution and examples of genes with multiple DIRs. **(A)** Venn diagrams showing intersect between super stress-responsive genes with associated multiple differential intron retention events across all stress treatments. The gene lists for short term and prolonged treatments for all tissue types (within each treatment) were pooled together to produce four combined lists of DIR-associated genes for drought, salt, heat, and cold treatments. **(B)** An example of the discrete stress-defined variations of multiple DIR events in a super stress-responsive gene. *POTRI.001G088800* gene encodes a homolog of *Arabidopsis* DCD (DEVELOPMENT AND CELL DEATH) domain protein (ptDCD-L). *Y*-axis represents the log of normalized intron coverage by RNA-Seq reads. **(C)** Iso-Seq models of transcript isoforms of *ptdcd-l* mRNA (*POTRI*.001G088800) under normal conditions and under combined abiotic stresses. **(D)** Multiple stress-responsive DIRs in the poplar mRNA encoding ptGAL (GLUTAMATE AMMONIA LIGASE, *POTRI.004G085400*). The top panel in **(D)** shows Iso-Seq models and individual single molecule reads of *ptgal* mRNA isoforms. The bottom panel shows stress-induced increase or decrease of statistically significant (*P*_adj_ < 0.05) DIRs in *ptgal* mRNA in leaf. Note that the retention of the sixth intron either increases, decreases, or remains unchanged in a stress type-specific manner. *Y*-axis shows the adjusted log fold change of normalized intron coverage by RNA-Seq reads. The numbering of introns and DIRs is depicted in black and red, respectively. Initial alignments of Iso-Seq reads to poplar genome for plotting on GMOD GBrowse were produced using STAR aligner V.2.5.2a (Dobin et al., 2013). with the following parameters: –runMode alignReads –outSAMattributes NH HI NM MD –readNameSeparator space –outFilterMultimapScoreRange 1 –outFilterMismatchNmax 2000 –scoreGapNoncan -20 –scoreGapGCAG -4 –scoreGapATAC -8 –scoreDelOpen -1 –scoreDelBase -1 –scoreInsOpen -1 –scoreInsBase -1 –alignEndsType Local –seedSearchStartLmax 50 –seedPerReadNmax 100000 –seedPerWindowNmax 1000 –alignTranscriptsPerReadNmax 100000 –alignTranscriptsPerWindowNmax 10000 –runThreadN 10. Iso-Seq models were generated using Transcriptome Analysis Pipeline for Isoform Sequencing (TAPIS) software (Abdel-Ghany et al., 2016).

**FILE 15 |** Lists of genes associated with multiple DIRs for each specific tissue/treatment.

**FILE 16 |** Intersect lists of genes associated with super-responsive DIRs.

**FILE 17 |** Stress-inducible DIR in poplar mRNA encoding heat stress transcription factor ptHSFA2. **(A)** iDiffIR models and RNA-Seq reads coverage of *pthsfA*2 mRNA. PCE designates: a poison cassette exon (e.g., an alternative exon introducing premature stop codon). Interestingly, this PCE event in *pthsfA*2 mRNA conserved similar to described previously PCE in *Arabidopsis hsfA*2 homolog (Filichkin et al., 2015a). *Y*-axis indicates the log of normalized intron coverage by RNA-Seq reads. **(B)** Iso-Seq models and individual cDNA reads. DIR event indicated by arrow, PCE – by asterisk. iDiffIR models were generated using iDiffIR (Xing et al., 2015) and SpliceGrapher (Rogers et al., 2012) software packages as described in Section “Materials and Methods.”

**FILE 18 |** Stress-induced DIR in *ptLEA* mRNA. *ptlea* (*POTRI*.*002G165000*) is an ortholog of *Arabidopsis late embryogenesis abundant 27* (*At2g46140*), which is a key gene involved in dehydration stress response and embryo development. **(A)** Adjusted log fold change of intron coverage across stress treatments and tissues. Drought, cold, and salt stresses decreased retention of the single *ptlea* intron in all tissues. However, heat stress increased IR in roots whereas there were no significant changes under heat in all other tissues. Note that the retention of the *ptlea* intron increases or decreases in roots under prolonged heat or cold stresses, respectively. It is also decreases consistently under heat, cold salt, and drought stresses in all other tissue types. Therefore, similar to multiple DIRs the retention of a single intron can be modulated in stress- and/or tissue-specific manner. *Y*-axis shows adjusted log fold change of normalized intron coverage by RNA-Seq reads. **(B)** Graphical output of iDiffIR showing normalized coverage of a DIR event in *ptlea* mRNA Y-axis shows the log of normalized intron coverage by RNA-Seq reads. DIR event shown in red.

**FILE 19 |** Stress-inducible DIR in mRNA encoding a poplar protein homologous to GALACTURONOSYLTRANSFERASE-LIKE and IRREGULAR XYLEM protein families (*POTRI.005G218900*). Note that retention of the first intron by heat stress occurs in xylem only suggesting a tissue specificity of this event. Graphical output of iDiffIR software showing normalized coverage of a DIR event (depicted in red). *Y*-axis: the log of normalized intron coverage.

**FILE 20 |** Stress-inducible DIR in transcripts of poplar homologs of the key regulators of plant circadian oscillator *ptca1/lhy-l* **(A)** and *ptgrp7-l* **(B)**. IR events in *ptcca1/lhy* (*circadian clock associated1/late elongated hypocotyl-like*; *POTRI.002G180800*) and *ptgrp7* (*glycine rich protein7-like*, *POTRI.009G116400*) mRNAs were differentially regulated by the temperature. This observation is consistent with our previous findings of regulation of similar intron retention events in *Arabidopsis* and *Brachypodium CCA1/LHY* homologs (Filichkin et al., 2010; Filichkin and Mockler, 2012; Filichkin et al., 2015a).

**FILE 21 |** An example of a cluster of transcripts containing stress co-regulated differential intron retention events (DIRs). Iso-Seq models show mRNAs encoding 40 KDA HEAT SHOCK PROTEIN (*POTRI.010g036200*) **(A)**, SERINE PROTEASE (*POTRI.012G077900*) **(B)**, DYNAMIN (*POTRI.017G041800*) **(C)**, and SAICAR SYNTHASE (*POTRI.017G051500*) **(D)**. The transcript features including all DIRs identified by iDiffIR software (see main **Figure 3**) consistent with the Iso-Seq models and individual reads. Notably, all DIR events in this cluster are located in the 3′ ends of mRNAs. The retention of introns in all four mRNAs will produce unusually long 3′ untranslated regions potentially targeting these transcripts for degradation by the nonsense mediated mRNA decay pathway.

**FILE 22 |** Iso-Seq models generally support transcript features (including differentially retained introns) predicted by the iDiffIR software using RNA-Seq data. Iso-Seq-predicted models of poplar *oncogene-related protein 2-like* (*ptOCR2-L*) **(A)** and *general transcription factor* II B (*ptTFIIB*) **(B)** mRNAs. **(C)** Coverage of *ptTFIIB* mRNA by RNA-Seq reads under normal conditions and high/low temperature stresses. Image generated using Poplar Interactome project web site (http://poplar.cgrb.oregonstate.edu/cgi-bin/gb2/gbrowse/poplar/). Retention of the 1st and 6th introns was quantified using reverse transcription – droplet digital PCR ((RT-ddPCR) as described in **Figure 5** and Section “Materials and Methods.”

**FILE 23 |** Splicing ratios of all introns in leaf across all stress treatments.

**FILE 24 |** Splicing ratios of all introns in root across all stress treatments.

**FILE 25 |** Splicing ratios of all introns in xylem across all stress treatments.

**FILE 26 |** Distribution of genes with co-regulated clusters of DIRs across all tissues and stress treatments.

**FILE 27 |** Moderate range temperature stress-driven increase of fully spliced *pttfIIB* mRNA (encodes GENERAL TRANSCRIPTION FACTOR II B) is accompanied by moderate increase of the relative ratio of the 1st intron-retaining isoform (I1R). **(A)** A time course of accumulation of fully spliced *pttfIIB* mRNA copies during increasing (heat stress) or decreasing (cold stress) temperature. **(B)** A time course of accumulation of the first intron-retaining *pttfIIB* mRNA during high or low temperature stresses. Absolute copy number of each isoform was determined using cDNA from reverse transcription reaction followed by the droplet digital PCR (RT-ddPCR) as described in Section “Materials and Methods.” RT-ddPCR of the fully spliced *pttfIIB* mRNA and its first intron-retaining isoform was performed using event-specific primers as described in Section “Materials and Methods.” **(C)** Relative ratio of the first intron-retaining isoform to its fully spliced counterpart mostly decreases with the increase of copy number of spliced mRNA and vice versa. Note that during heat stress treatment segment the level of spliced mRNA shows limited changes whereas relative proportion of I1R isoform increased substantially. Young poplar plants were heat treated (42°C) for 24 h followed by the transition to cold temperature (4°C) for another 24 h as described in Section “Materials and Methods.” Diagram at the bottom shows fluctuations of temperature during the time course of treatment. Each time point represents 2 h.

**FILE 28 |** Examples of extensive alternative splicing and multiple intron retention events in primary transcripts of poplar miRNAs ptc-miR156e and ptc-miR398c. Numbers in gray boxes indicate genomic coordinates of splice junctions.

**FILE 29 |** Normalized gene expression values (RNA-seq reads) across all stress treatments.
