# Supplementary material for: Abiotic Stresses Modulate Landscape of Poplar Transcriptome via Alternative Splicing, Differential Intron Retention, and Isoform Ratio Switching
Source: Front Plant Sci. 2018 Feb 12;9:5. doi: 10.3389/fpls.2018.00005 (PMC5816337; doi:10.3389/fpls.2018.00005)
Supplement: Supplementary file 1 [file Data_Sheet_1.zip › Supplementary file 1-16/Supplementary File 1.pdf]

A

SAMPLING STRATEGY, STRESS TREATMENTS AND PREPARATION OF RNA-Seq and Iso-Seq LIBRARIES

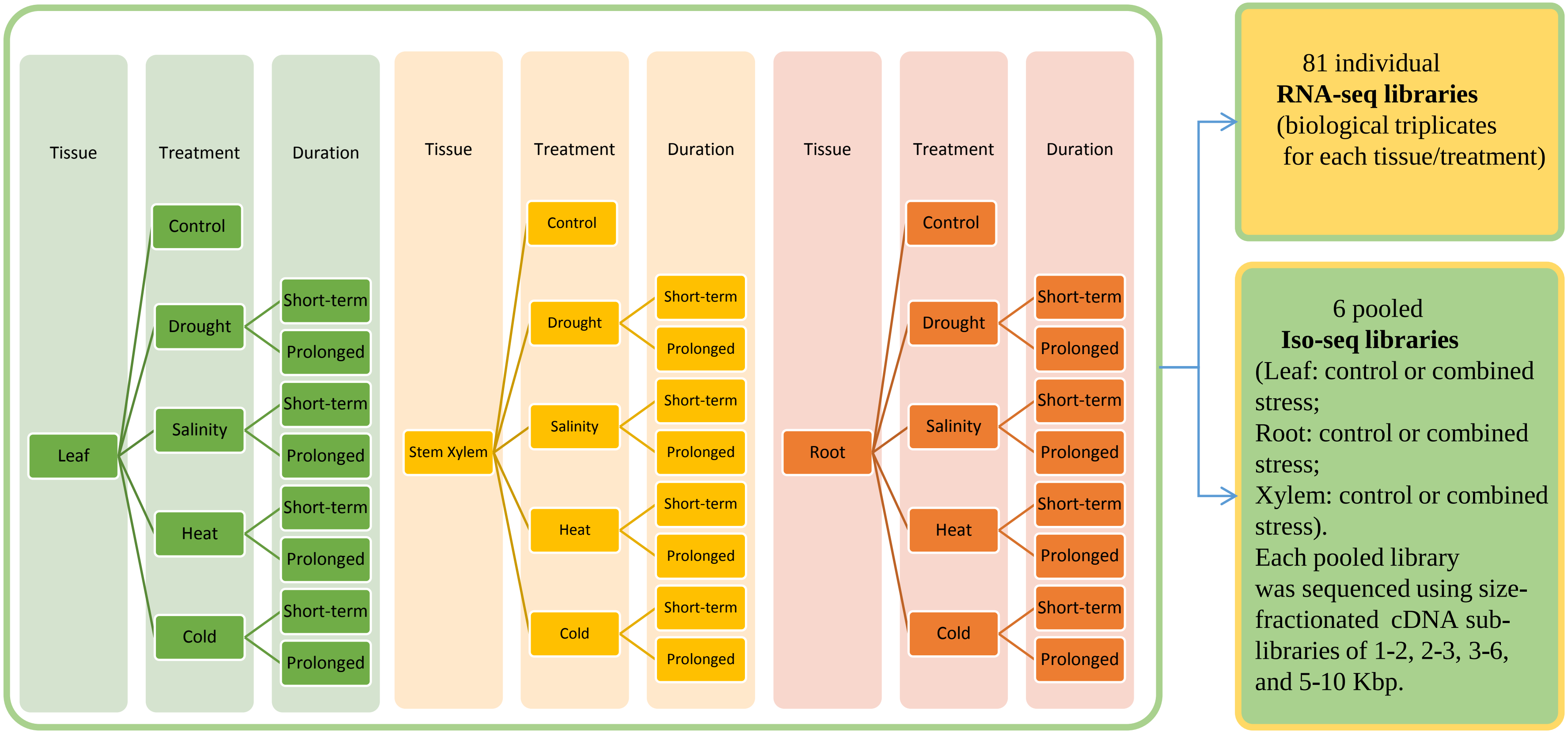

Supplementary File 1A. Sampling strategy and flow of data analysis. (A) Sample collection strategy and consequent steps of data analyses to detect and validate DIR events.

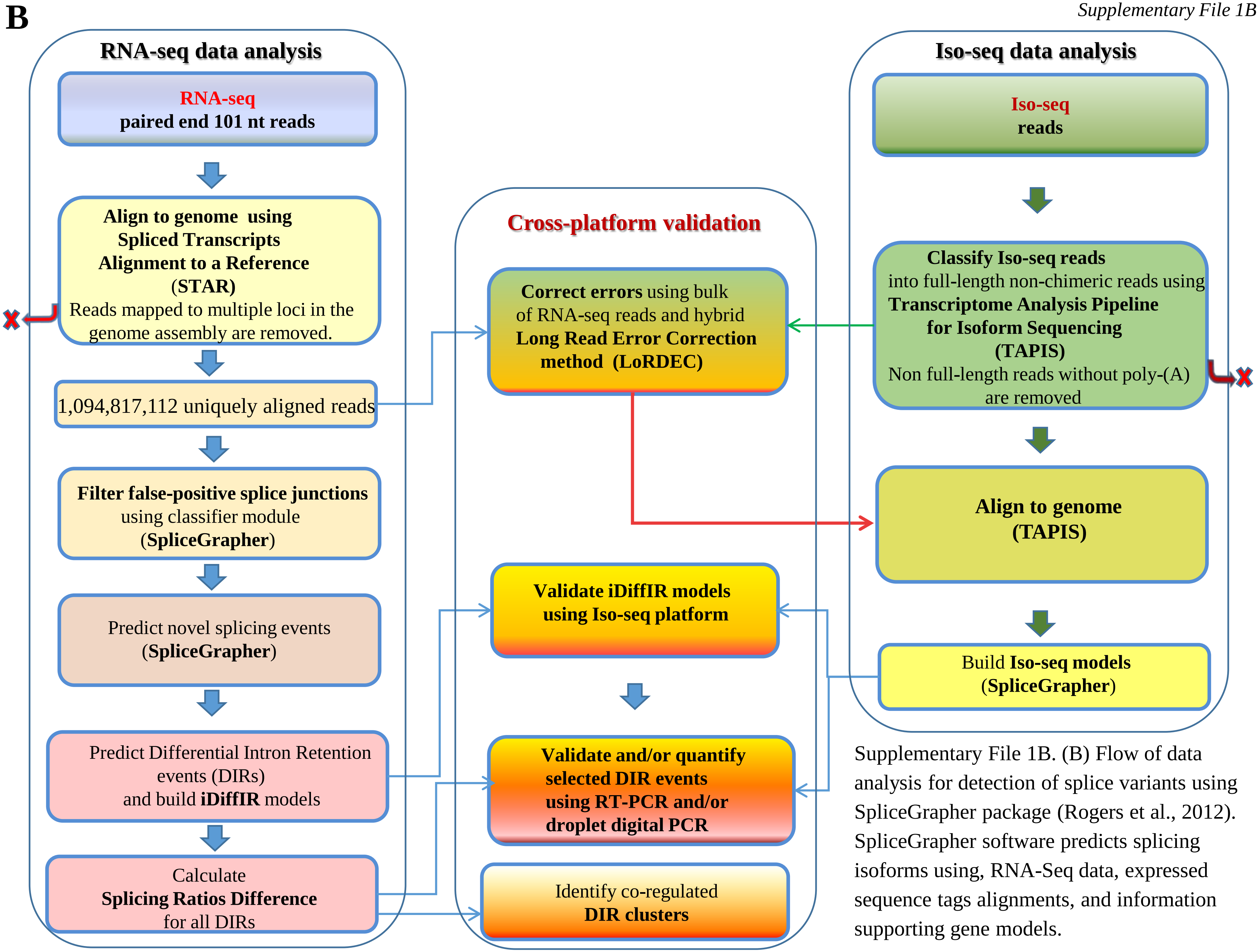

Supplementary File 1B. (B) Flow of data analysis for detection of splice variants using SpliceGrapher package (Rogers et al., 2012). SpliceGrapher software predicts splicing isoforms using, RNA-Seq data, expressed sequence tags alignments, and information supporting gene models.
