# Supplementary material for: Abiotic Stresses Modulate Landscape of Poplar Transcriptome via Alternative Splicing, Differential Intron Retention, and Isoform Ratio Switching
Source: Front Plant Sci. 2018 Feb 12;9:5. doi: 10.3389/fpls.2018.00005 (PMC5816337; doi:10.3389/fpls.2018.00005)
Supplement: Supplementary file 1 [file Data_Sheet_1.zip › Supplementary file 1-16/Supplementary File 10.pdf]

ABA responsive element binding factor (*AREB* gene family)

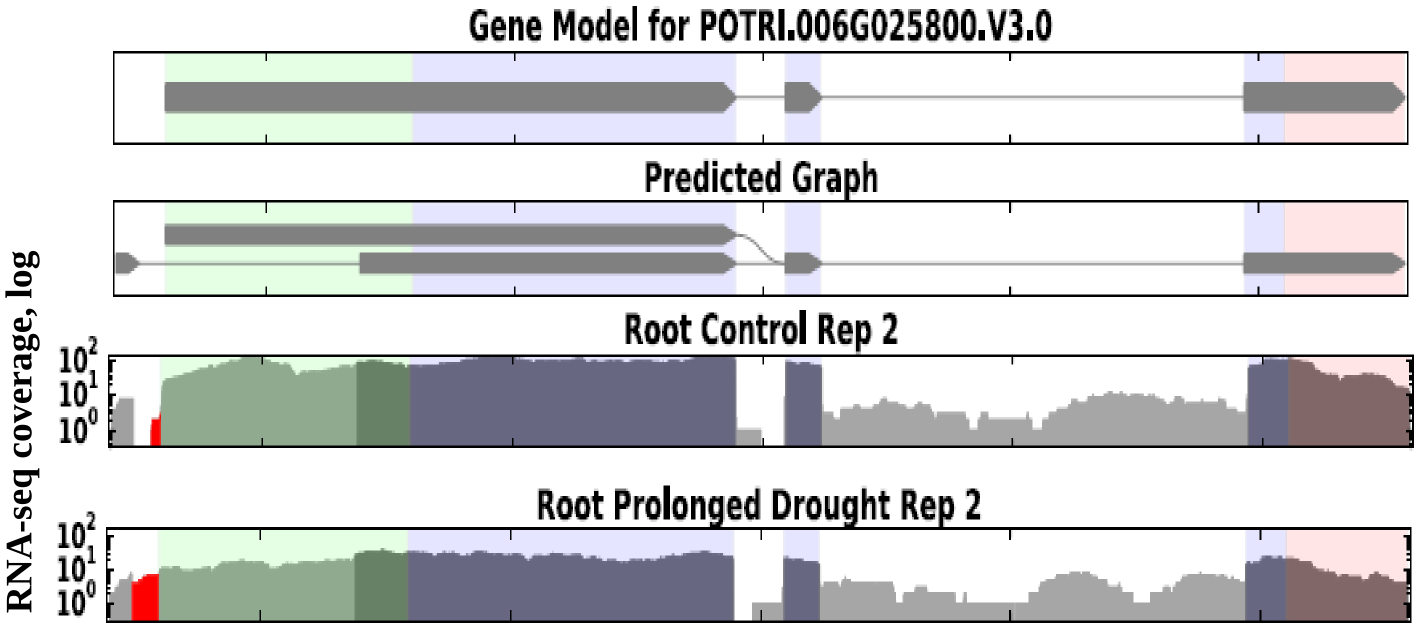

dehydration responsive element binding (DREB gene family)

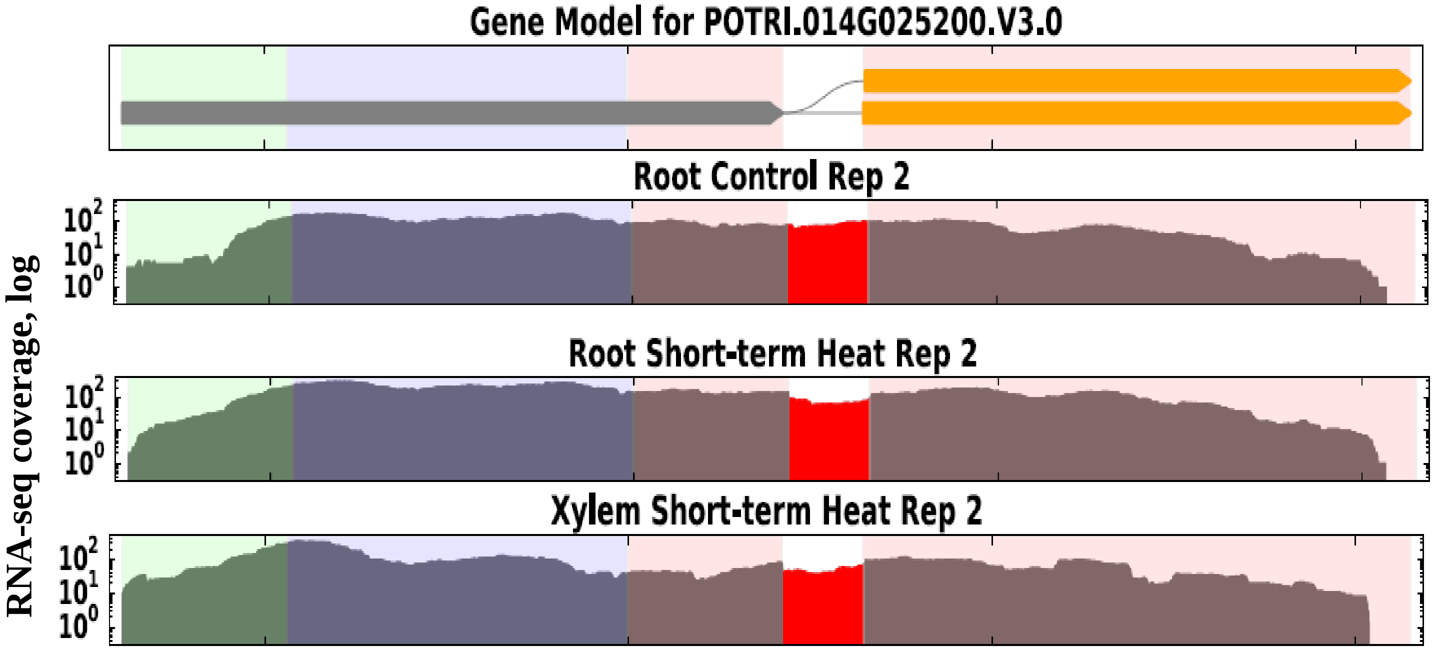

Supplementary File 10. Examples of stress-inducible DIRs in mRNAs of *ABA-responsive element binding factors (AREB) and dehydration-responsive element binding (DREB)* gene families. Y-axis shows the log of normalized intron coverage.
