# Supplementary material for: Abiotic Stresses Modulate Landscape of Poplar Transcriptome via Alternative Splicing, Differential Intron Retention, and Isoform Ratio Switching
Source: Front Plant Sci. 2018 Feb 12;9:5. doi: 10.3389/fpls.2018.00005 (PMC5816337; doi:10.3389/fpls.2018.00005)
Supplement: Supplementary file 1 [file Data_Sheet_1.zip › Supplementary file 1-16/Supplementary File 13.pdf]

# DIR-associated genes: an intersect between short- and long- term treatments

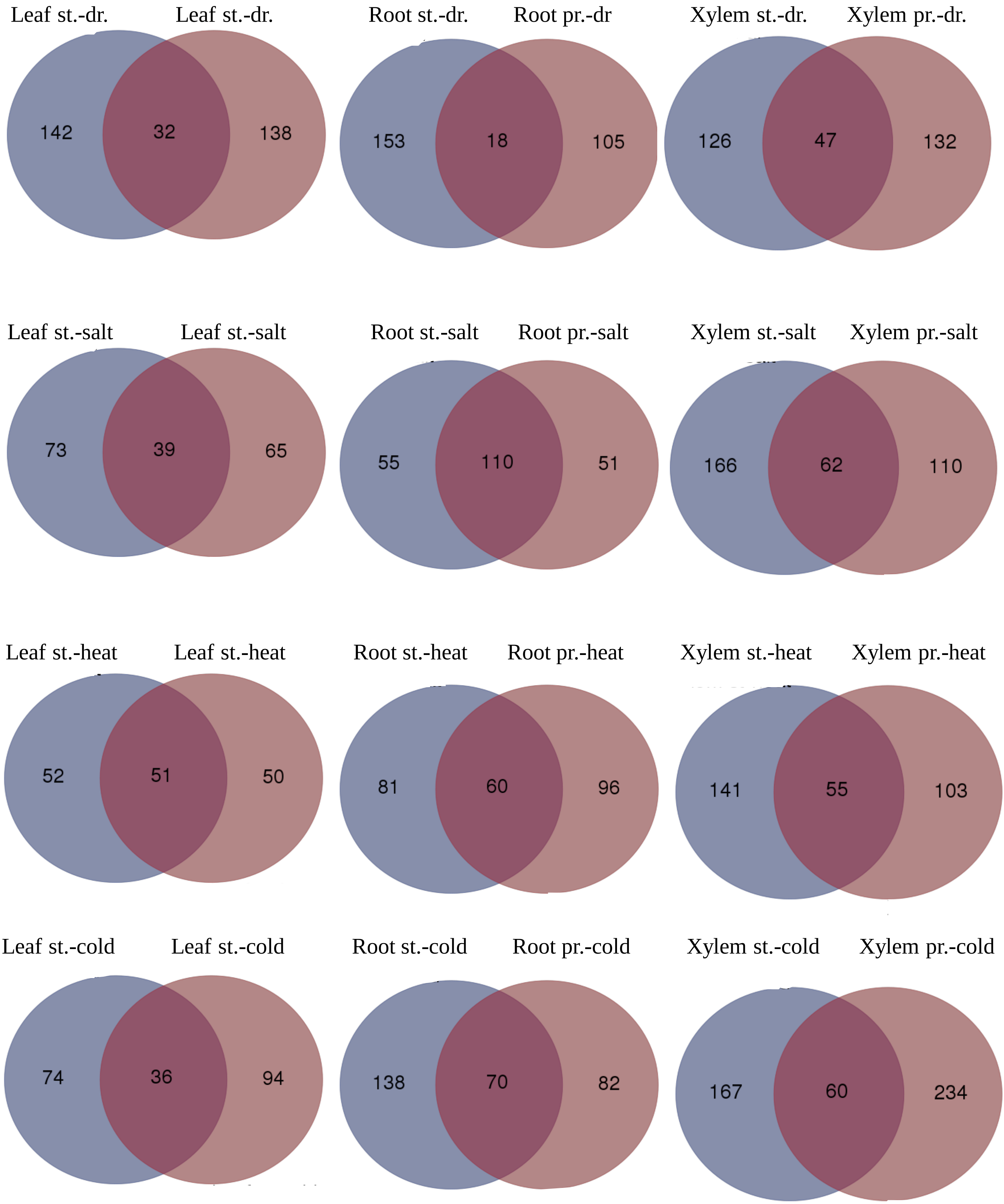


Supplementary File 13. Venn diagrams representation of overlaps between the genes associated with DIR events induced by short term or prolonged treatment phases.
