# Supplementary material for: Abiotic Stresses Modulate Landscape of Poplar Transcriptome via Alternative Splicing, Differential Intron Retention, and Isoform Ratio Switching
Source: Front Plant Sci. 2018 Feb 12;9:5. doi: 10.3389/fpls.2018.00005 (PMC5816337; doi:10.3389/fpls.2018.00005)

Supplementary File 14. Distribution and examples of genes with multiple DIRs. (a) Venn diagrams showing intersect between super stress-responsive genes with associated multiple differential intron retention events across all stress treatments. The gene lists for short term and prolonged treatments for all tissue types (within each treatment) were pooled together to produce four combined lists of DIR-associated genes for drought, salt, heat, and cold treatments. (b) An example of the discrete stress-defined variations of multiple DIR events in a super stress-responsive gene. *POTRI.001G088800* gene encodes a homolog of Arabidopsis DCD (DEVELOPMENT AND CELL DEATH) domain protein (ptDCD-L). Y-axis represents the log of normalized intron coverage by RNA-Seq reads. (c) Iso-Seq models of transcript isoforms of *ptdcd-l* mRNA (*POTRI*.001G088800) under normal conditions and under combined abiotic stresses. (d) Multiple stress-responsive DIRs in the poplar mRNA encoding ptGAL (GLUTAMATE AMMONIA LIGASE, *POTRI.004G085400*). The top panel in (d) shows Iso-Seq models and individual single molecule reads of *ptgal* mRNA isoforms. The bottom panel shows stress-induced increase or decrease of statistically significant (Padj <0.05) DIRs in *ptgal* mRNA in leaf. Note that the retention of the sixth intron either increases, decreases, or remains unchanged in a stress type-specific manner. Y-axis shows the adjusted log fold change of normalized intron coverage by RNA-Seq reads. The numbering of introns and DIRs is depicted in black and red respectively. Initial alignments of Iso-Seq reads to poplar genome for plotting on GMOD GBrowse were produced using STAR aligner V.2.5.2a (Dobin et al., 2013). with the following parameters: --runMode alignReads --outSAMattributes NH HI NM MD --readNameSeparator space --outFilterMultimapScoreRange 1 --outFilterMismatchNmax 2000 --scoreGapNoncan -20 --scoreGapGCAG -4 --scoreGapATAC -8 --scoreDelOpen -1 --scoreDelBase -1 --scoreInsOpen -1 --scoreInsBase -1 --alignEndsType Local --seedSearchStartLmax 50 --seedPerReadNmax 100000 --seedPerWindowNmax 1000 --alignTranscriptsPerReadNmax 100000 --alignTranscriptsPerWindowNmax 10000 --runThreadN 10. Iso-Seq models were generated using Transcriptome Analysis Pipeline for Isoform Sequencing (TAPIS) software (Abdel-Ghany *et al.*, 2016).

A

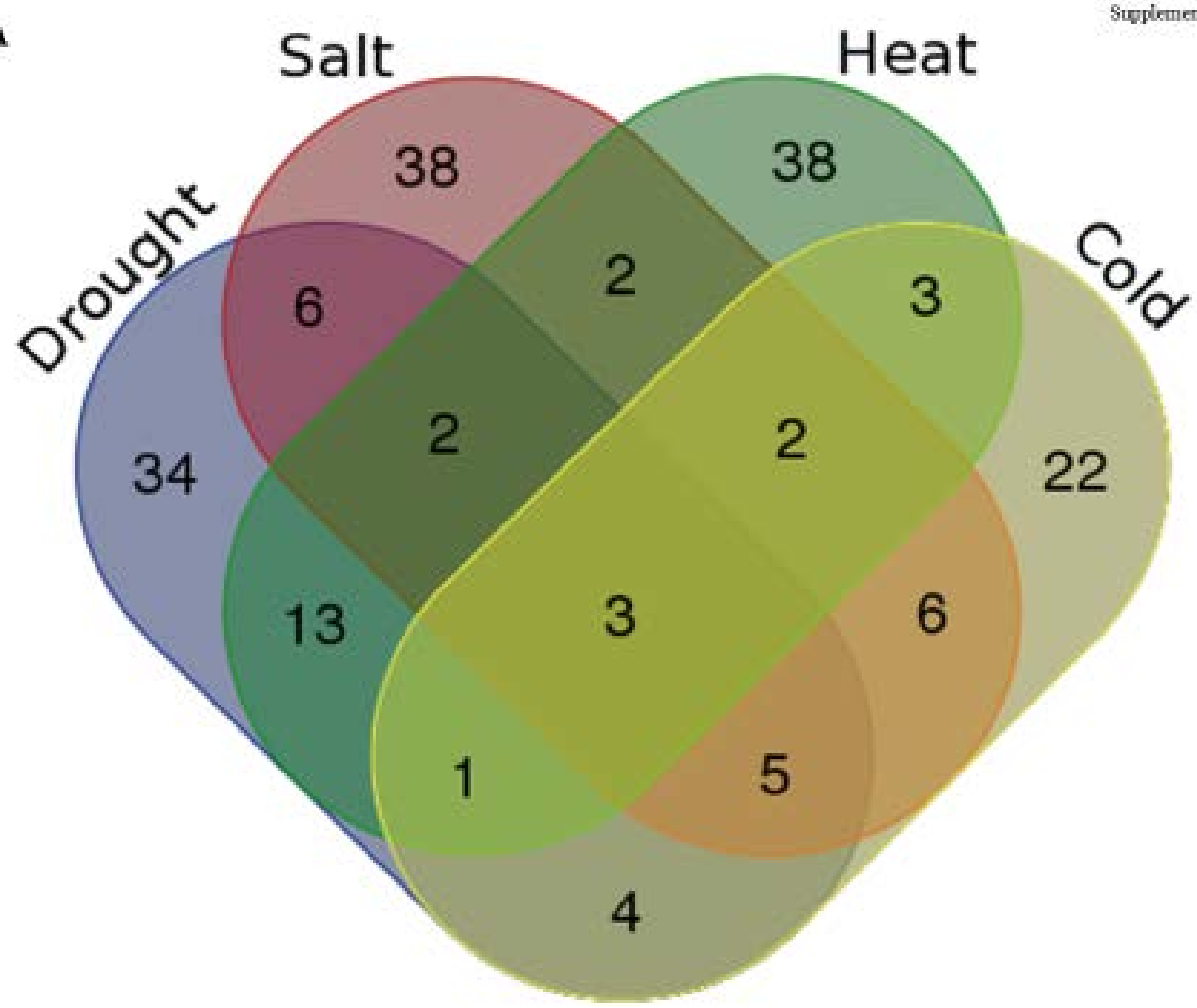

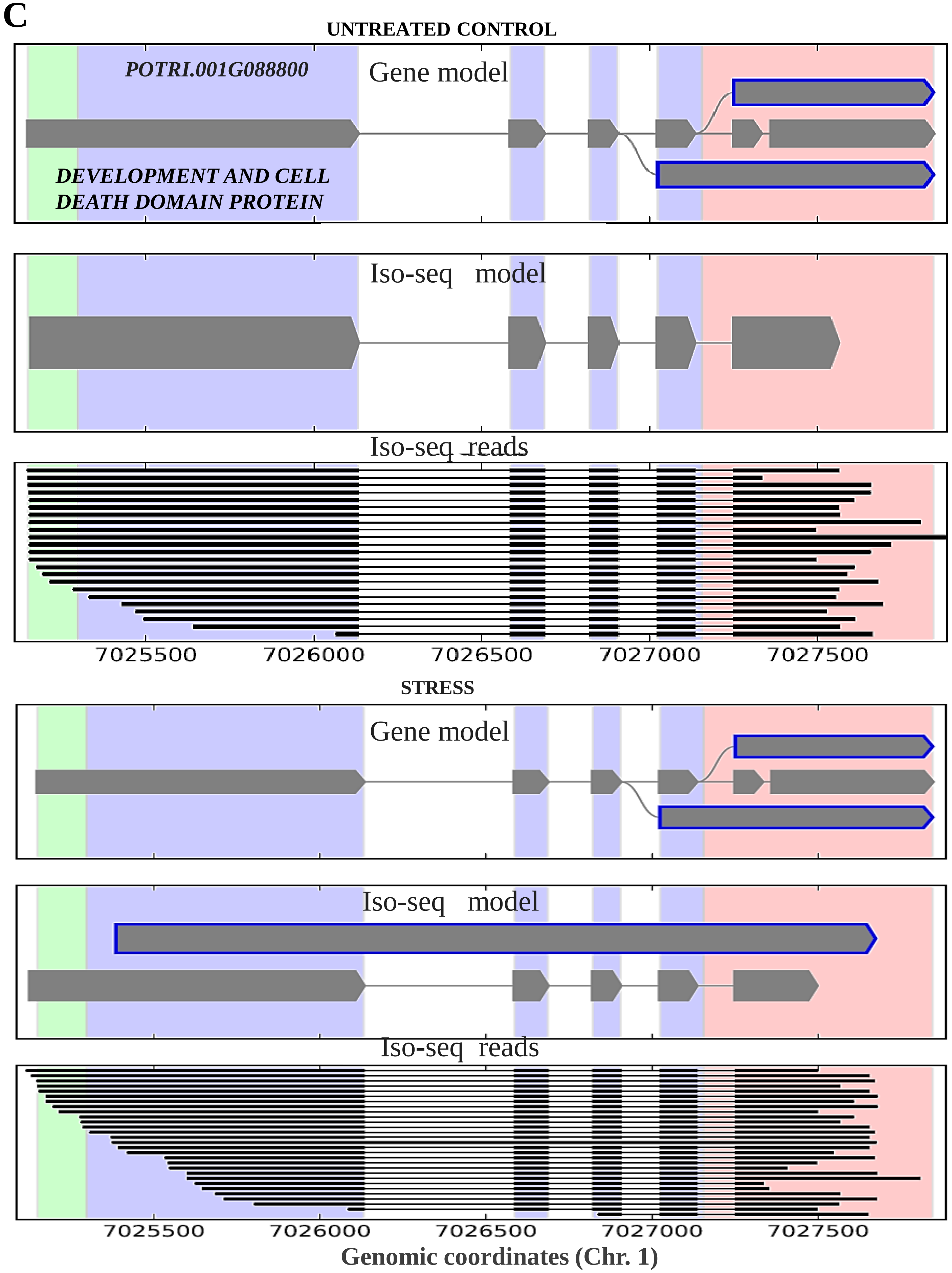
C
UNTREATED CONTROL
POTRI.001G088800
Gene model
DEVELOPMENT AND CELL
DEATH DOMAIN PROTEIN
Iso-seq model
Iso-seq reads
7025500
7026000
7026500
7027000
7027500
STRESS
Gene model
Iso-seq model
Iso-seq reads
7025500
7026000
7026500
7027000
7027500
Genomic coordinates (Chr. 1)

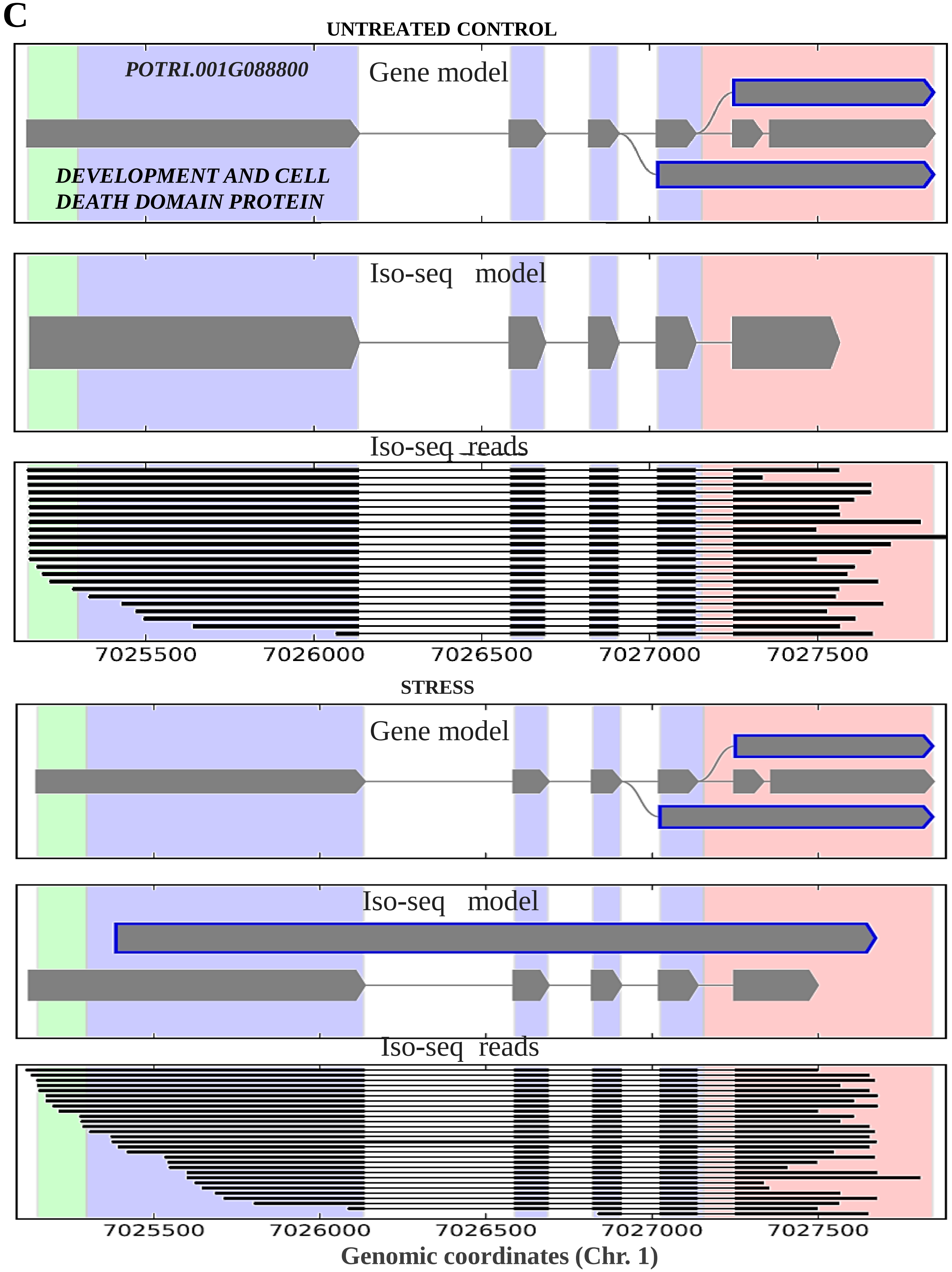
C
UNTREATED CONTROL
POTRI.001G088800
Gene model
DEVELOPMENT AND CELL
DEATH DOMAIN PROTEIN
Iso-seq model
Iso-seq reads
7025500
7026000
7026500
7027000
7027500
STRESS
Gene model
Iso-seq model
Iso-seq reads
7025500
7026000
7026500
7027000
7027500
Genomic coordinates (Chr. 1)

D

***glutamate ammonia ligase* transcript models**

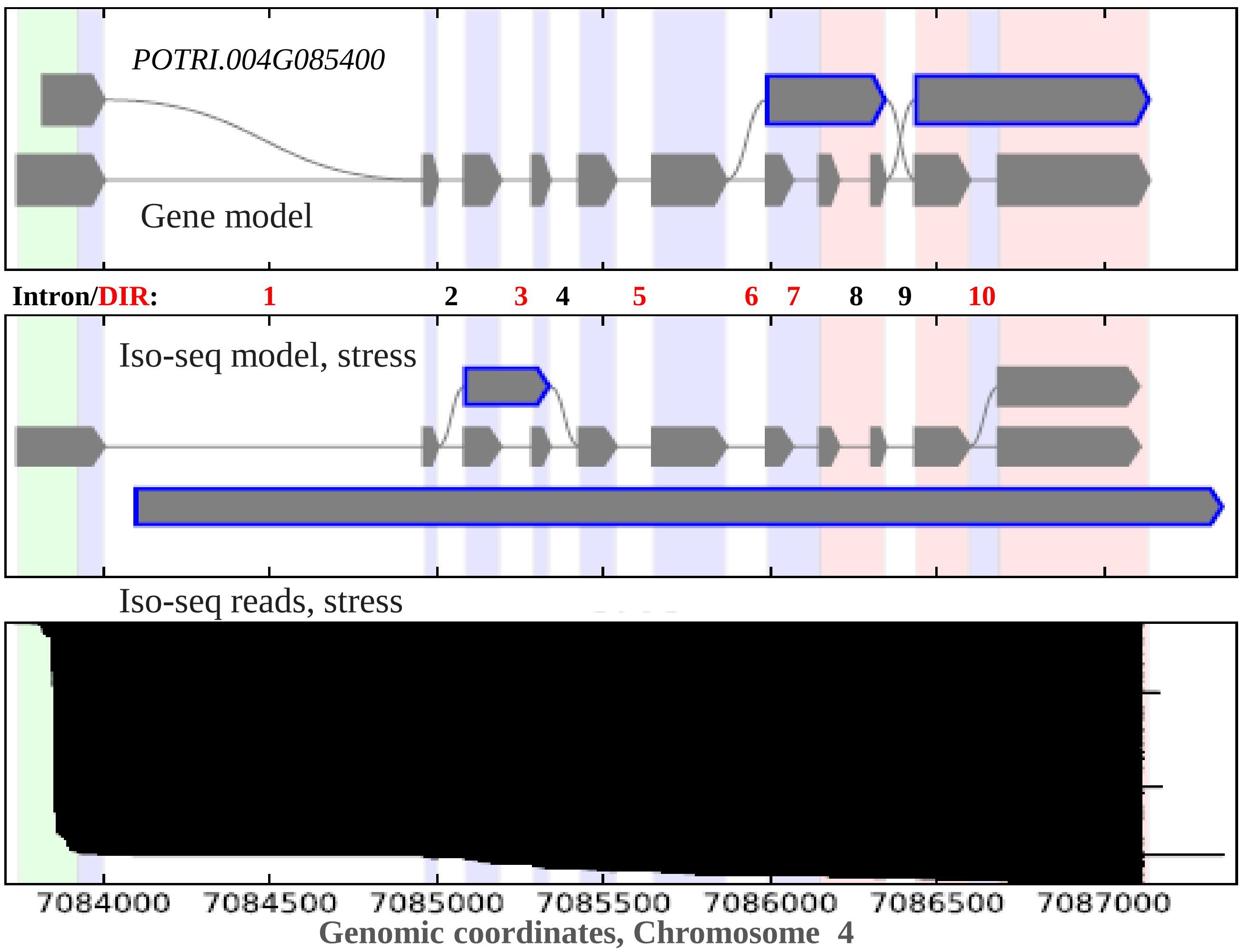

Stress-induced differential intron retention events in *glutamate ammonia ligase* mRNA in leaf

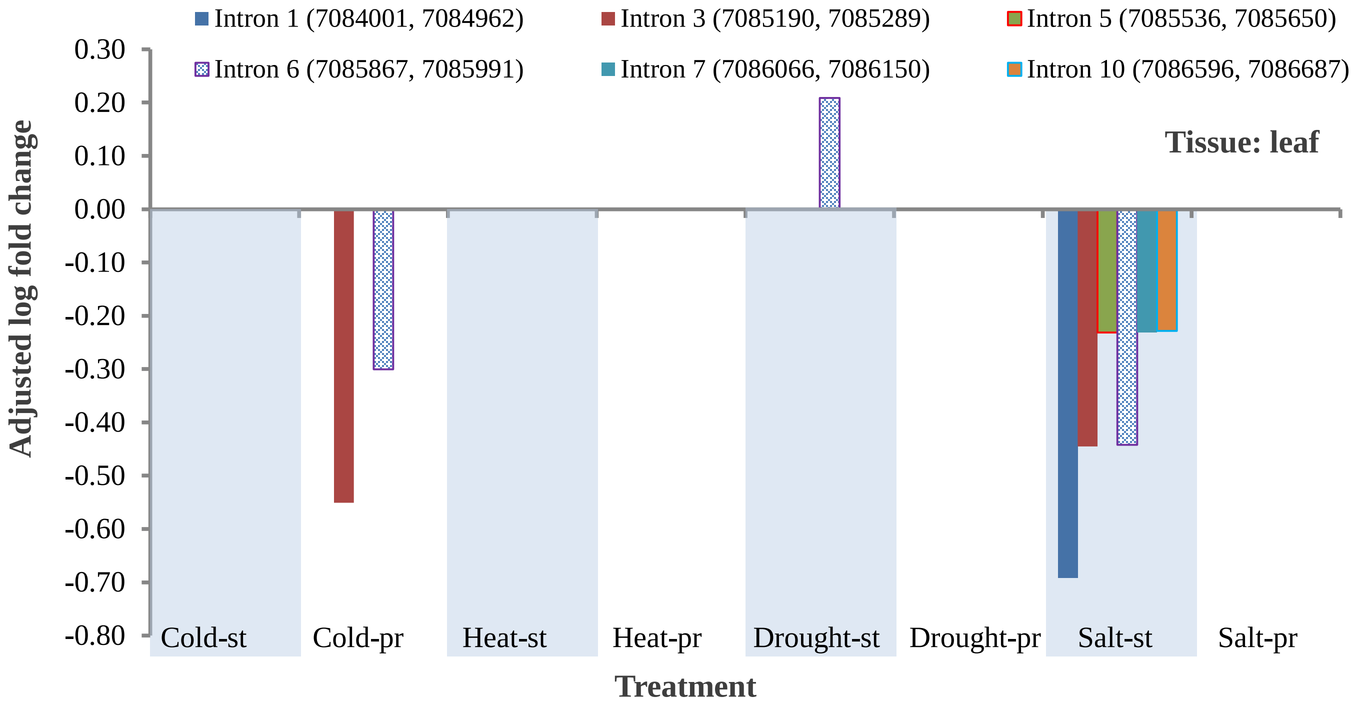

Supplement: Supplementary file 1 [file Data_Sheet_1.zip › Supplementary file 1-16/Supplementary File 14.pdf]
