# Supplementary material for: Abiotic Stresses Modulate Landscape of Poplar Transcriptome via Alternative Splicing, Differential Intron Retention, and Isoform Ratio Switching
Source: Front Plant Sci. 2018 Feb 12;9:5. doi: 10.3389/fpls.2018.00005 (PMC5816337; doi:10.3389/fpls.2018.00005)
Supplement: Supplementary file 1 [file Data_Sheet_1.zip › Supplementary file 1-16/Supplementary File 8.pdf]

A

B

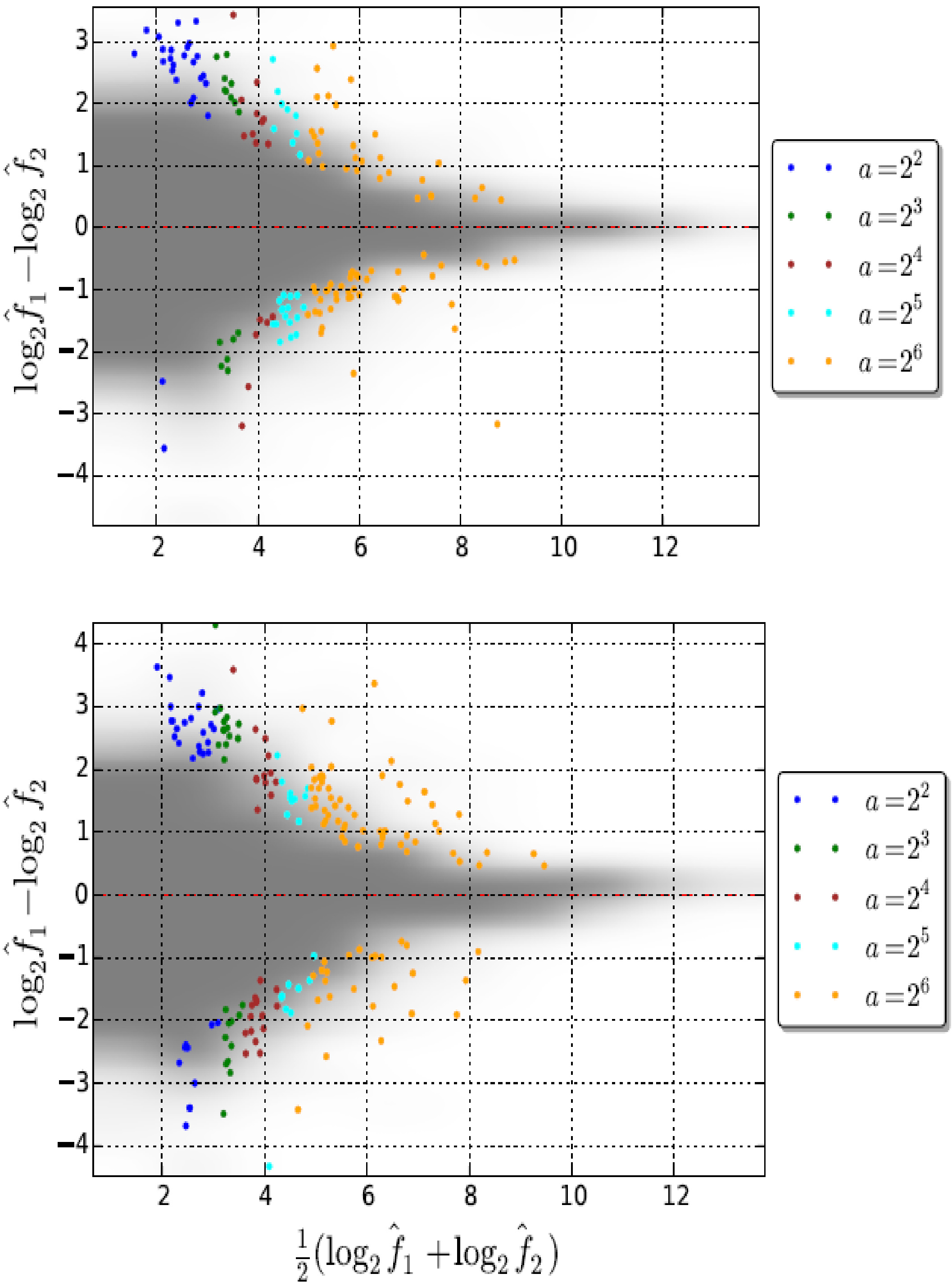

Supplementary File 8. The distribution of DIR events: typical examples of Multivariate analysis (MvA) plots. (A) Short term root heat stress. (B) Prolonged heat stress in root. Colored points represent intron retention events with statistically significant differential retention values (P*adj* equal or lesser 0.05).
