# Supplementary material for: Abiotic Stresses Modulate Landscape of Poplar Transcriptome via Alternative Splicing, Differential Intron Retention, and Isoform Ratio Switching
Source: Front Plant Sci. 2018 Feb 12;9:5. doi: 10.3389/fpls.2018.00005 (PMC5816337; doi:10.3389/fpls.2018.00005)
Supplement: Supplementary file 1 [file Data_Sheet_1.zip › Supplementary file 1-16/Supplementary File 9.pdf]

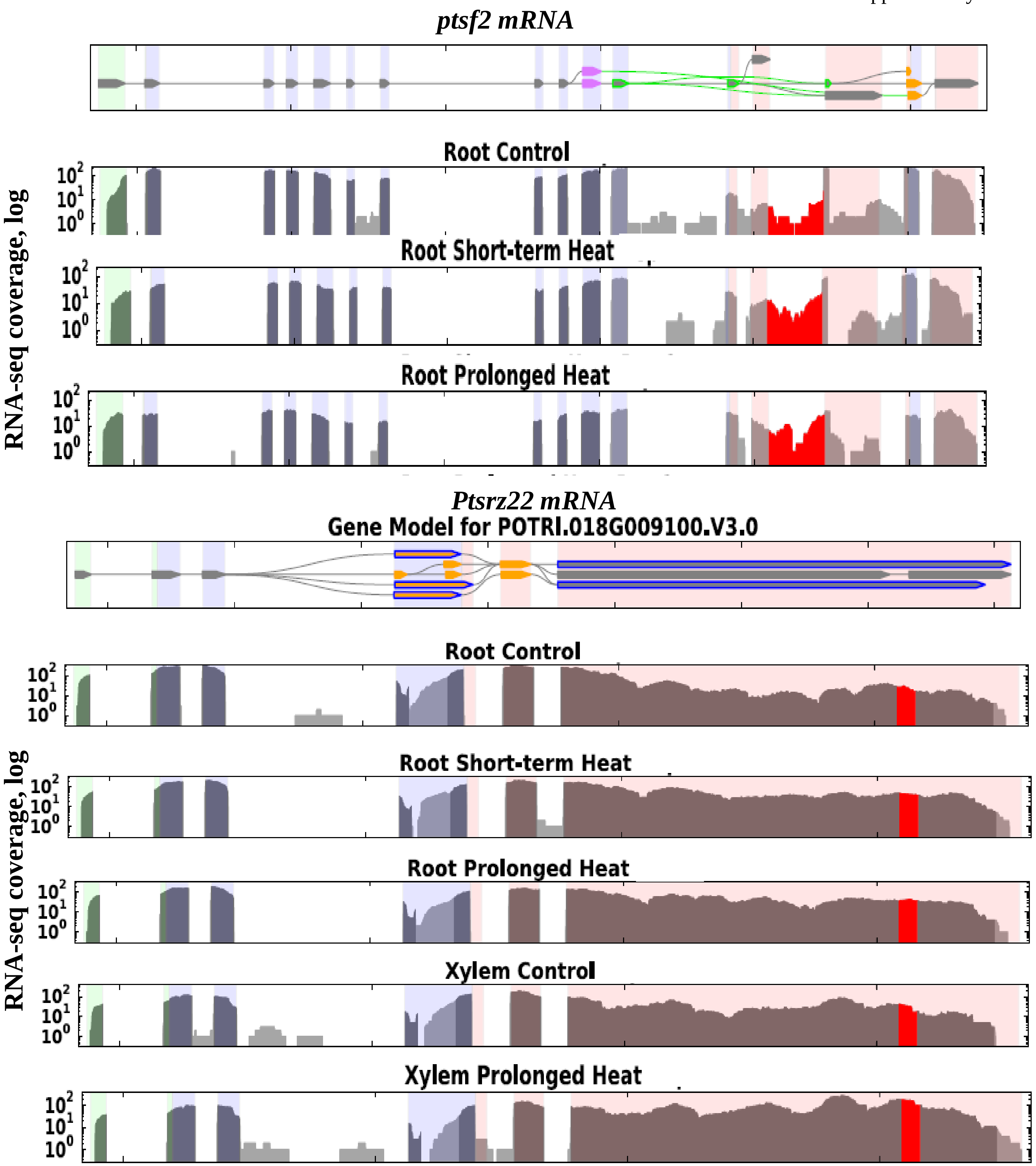

Supplementary File 9. Examples of temperature-inducible DIRs in transcripts encoding ptSF2 and ptSRZ22 splicing factors. ptSF2 (*POTRI.T134200*, top panel) is an orthologue of a human general/alternative splicing factor SF2/ASF SF2 and co-orthologue of Arabidopsis serine/arginine-rich proteins R34/SR1 (*At1g02840*) and SR34B (encoded by *At4g024302*). ptSRZ22 (*POTRI.018G009100*, bottom panel) is an orthologue of a mammalian 9G8 SF and the Arabidopsis serine/arginine-rich protein AthRSZp22 (*AT4G31580*). Y-axis shows the log of normalized intron coverage by RNA-Seq reads.
