# Supplementary figures and images for: Abiotic Stresses Modulate Landscape of Poplar Transcriptome via Alternative Splicing, Differential Intron Retention, and Isoform Ratio Switching
Source: Front Plant Sci. 2018 Feb 12;9:5. doi: 10.3389/fpls.2018.00005 (PMC5816337; doi:10.3389/fpls.2018.00005)

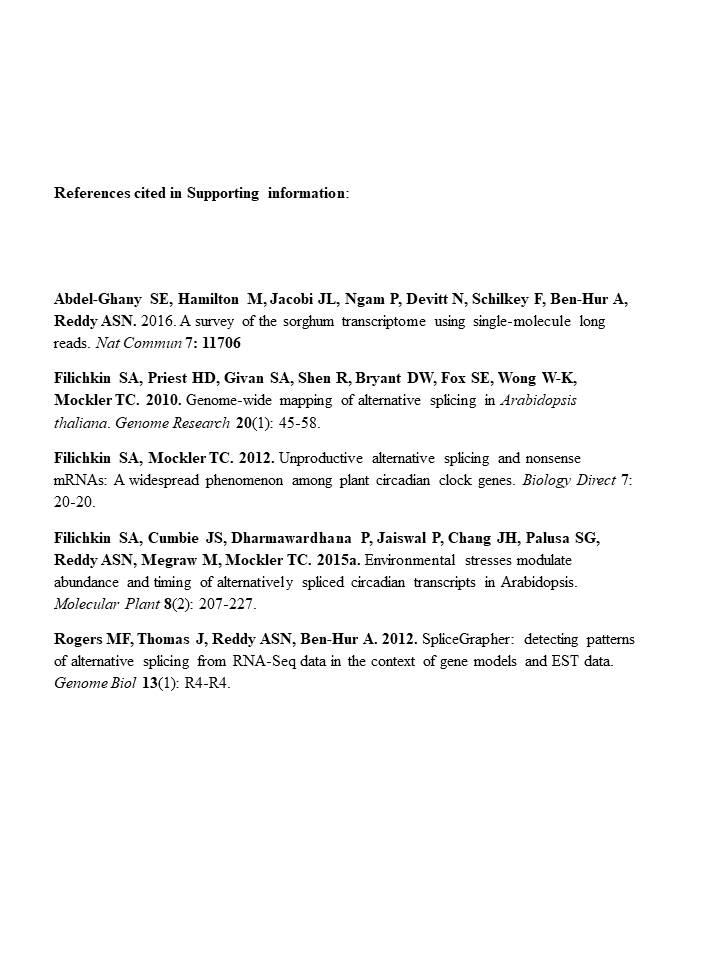

Supplement: Supplementary file 1 [file Data_Sheet_1.zip › Supplementary file 1-16/References cited in Supporting information.jpg]
