# Supplementary material for: Abiotic Stresses Modulate Landscape of Poplar Transcriptome via Alternative Splicing, Differential Intron Retention, and Isoform Ratio Switching
Source: Front Plant Sci. 2018 Feb 12;9:5. doi: 10.3389/fpls.2018.00005 (PMC5816337; doi:10.3389/fpls.2018.00005)

Supplementary File 17A-B_legend

Supplementary File 17. Stress-inducible DIR in poplar mRNA encoding heat stress transcription factor ptHSFA2. (A) iDiffIR models and RNA-Seq reads coverage of *pthsfA*2 mRNA. PCE designates: a poison cassette exon (e.g., an alternative exon introducing premature stop codon). Interestingly, this PCE event in *pthsfA*2 mRNA conserved similar to described previously PCE in Arabidopsis *hsfA*2 homolog (Filichkin *et al*., 2015a). Y-axis indicates the log of normalized intron coverage by RNA-Seq reads. (B) Iso-Seq models and individual cDNA reads. DIR event indicated by arrow, PCE – by asterisk. iDiffIR models were generated using iDiffIR (Xing et al., 2015) and SpliceGrapher (Rogers *et al*., 2012) software packages as described in Materials and Methods.

Supplementary File 17A-B

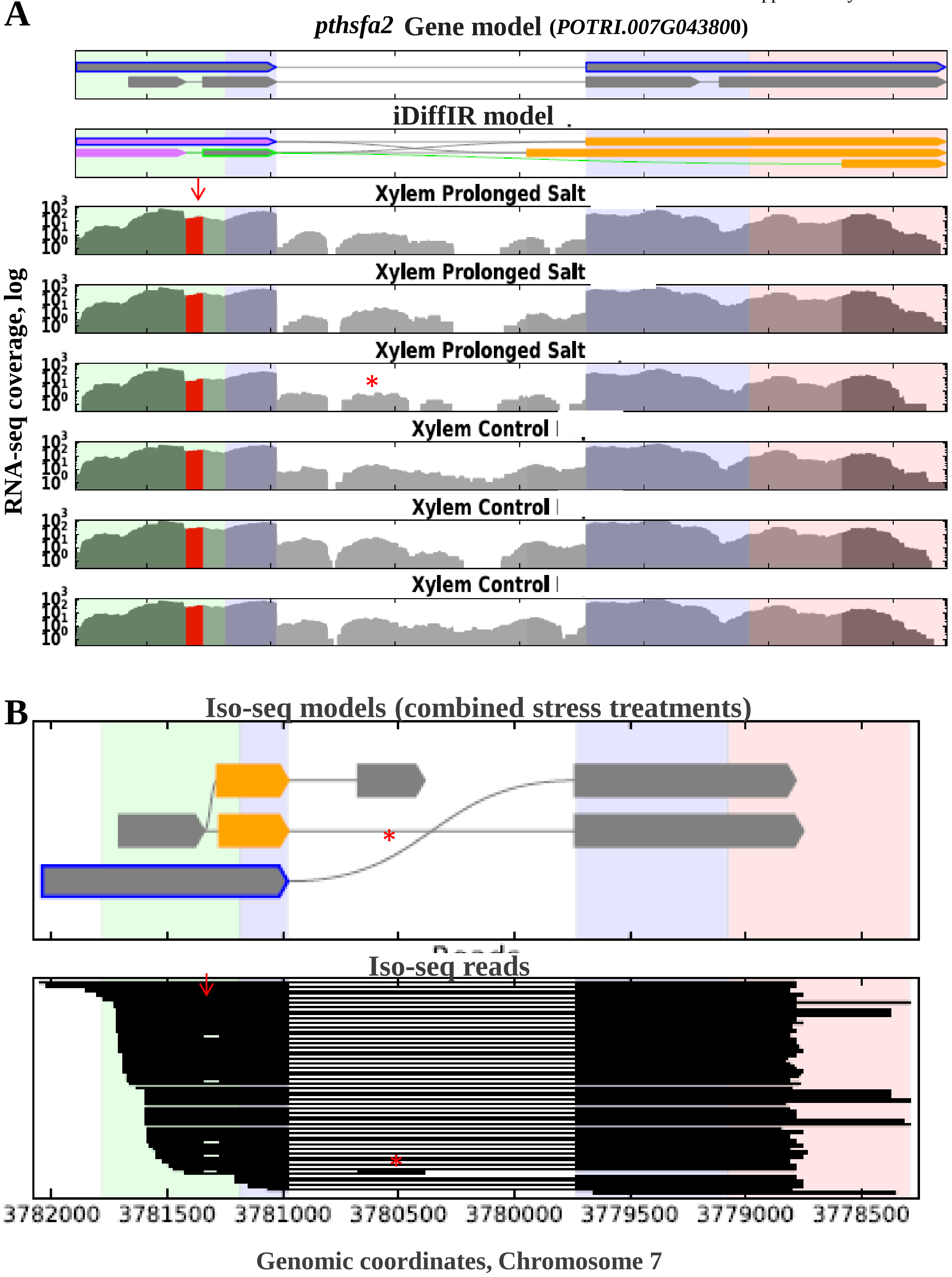

Supplement: Supplementary file 2 [file Data_Sheet_2.zip › Supplementary files 17-24/Supplementary File 17.pdf]
