# Supplementary material for: Abiotic Stresses Modulate Landscape of Poplar Transcriptome via Alternative Splicing, Differential Intron Retention, and Isoform Ratio Switching
Source: Front Plant Sci. 2018 Feb 12;9:5. doi: 10.3389/fpls.2018.00005 (PMC5816337; doi:10.3389/fpls.2018.00005)

Supplementary File 18. Stress-induced DIR in *ptlea* mRNA. *ptlea* (*POTRI.002G165000*) is an orthologue of Arabidopsis *late embryogenesis abundant 27* (*At2g46140*), which is a key gene involved in dehydration stress response and embryo development. (A) Adjusted log fold change of intron coverage across stress treatments and tissues. Drought, cold and salt stresses decreased retention of the single *ptlea* intron in all tissues. However, heat stress increased intron retention in roots whereas there were no significant changes under heat in all other tissues. Note that the retention of the *ptlea* intron increases or decreases in roots under prolonged heat or cold stresses, respectively. It is also decreases consistently under heat, cold salt, and drought stresses in all other tissue types. Therefore, similar to multiple DIRs the retention of a single intron can be modulated in stress- and/or tissue-specific manner. Y-axis shows adjusted log fold change of normalized intron coverage by RNA-Seq reads. (B) Graphical output of iDiffIR showing normalized coverage of a DIR event in *ptlea* mRNA Y-axis shows the log of normalized intron coverage by RNA-Seq reads. DIR event shown in red.

A

DIR event in poplar mRNA encoding LATE EMBRYOGENESIS ABUNDANT (*ptlea, POTRI.002G165000*)

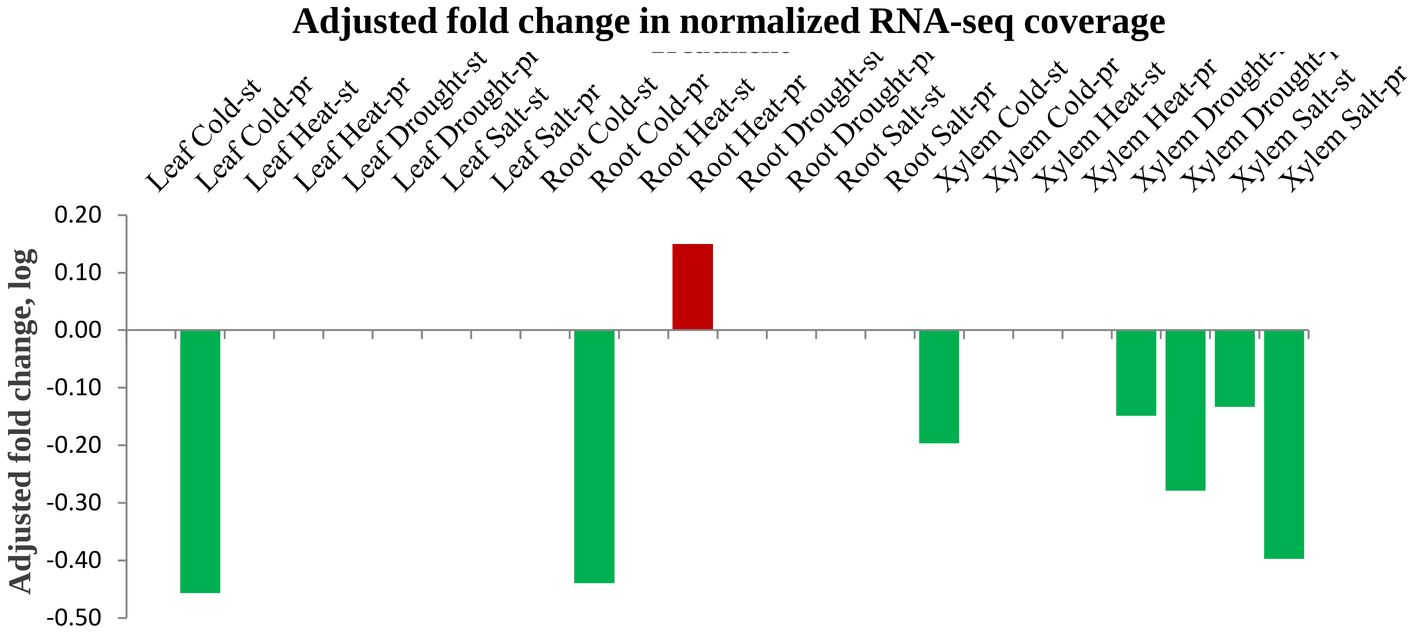

B

RNA-seq coverage of a single DIR in *ptlea* mRNA

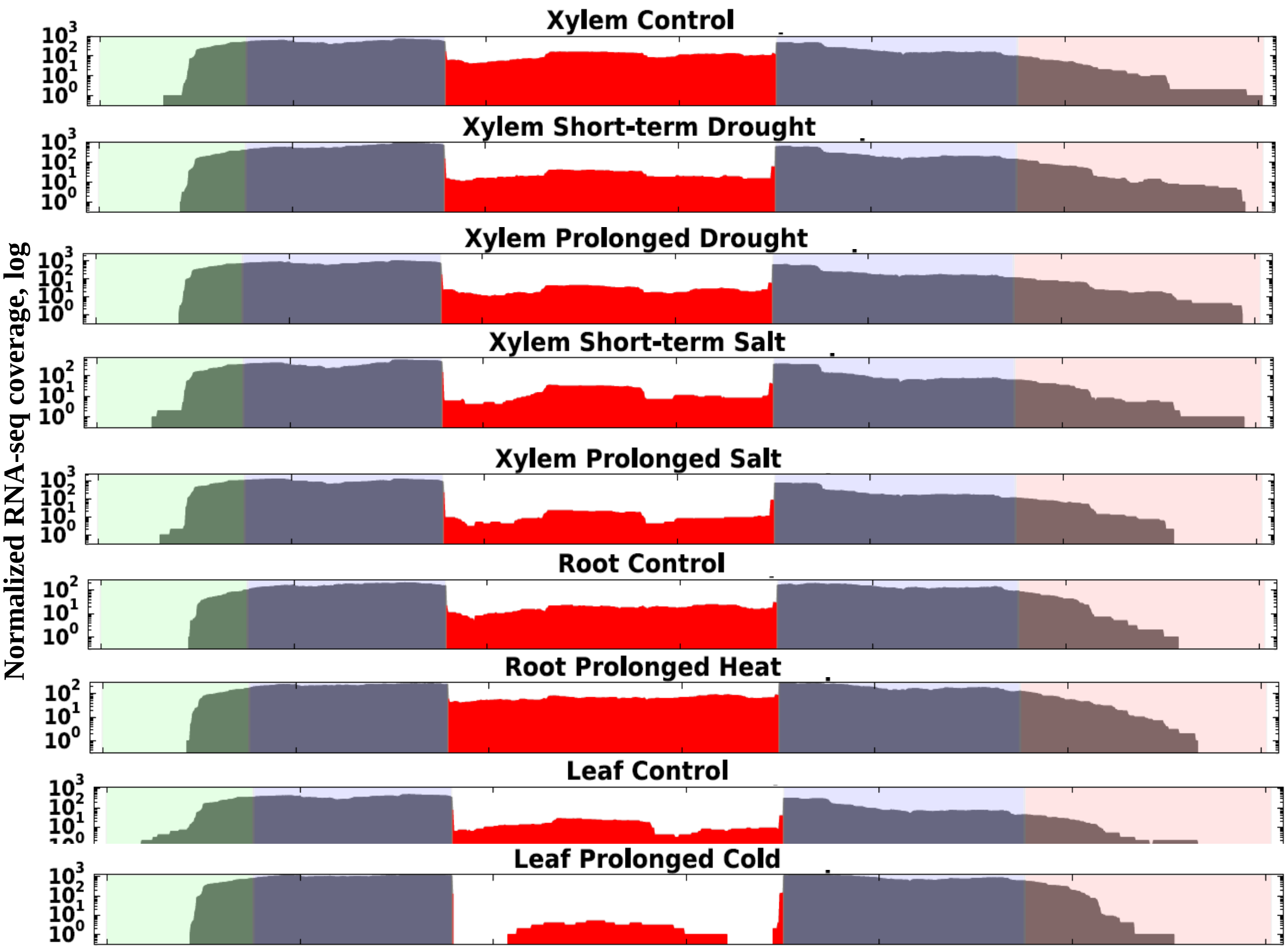

Supplement: Supplementary file 2 [file Data_Sheet_2.zip › Supplementary files 17-24/Supplementary File 18.pdf]
