# Supplementary material for: Abiotic Stresses Modulate Landscape of Poplar Transcriptome via Alternative Splicing, Differential Intron Retention, and Isoform Ratio Switching
Source: Front Plant Sci. 2018 Feb 12;9:5. doi: 10.3389/fpls.2018.00005 (PMC5816337; doi:10.3389/fpls.2018.00005)
Supplement: Supplementary file 2 [file Data_Sheet_2.zip › Supplementary files 17-24/Supplementary File 19.pdf]

mRNA encoding poplar GLYCOSYL TRANSFERASE FAMILY 8 PROTEIN

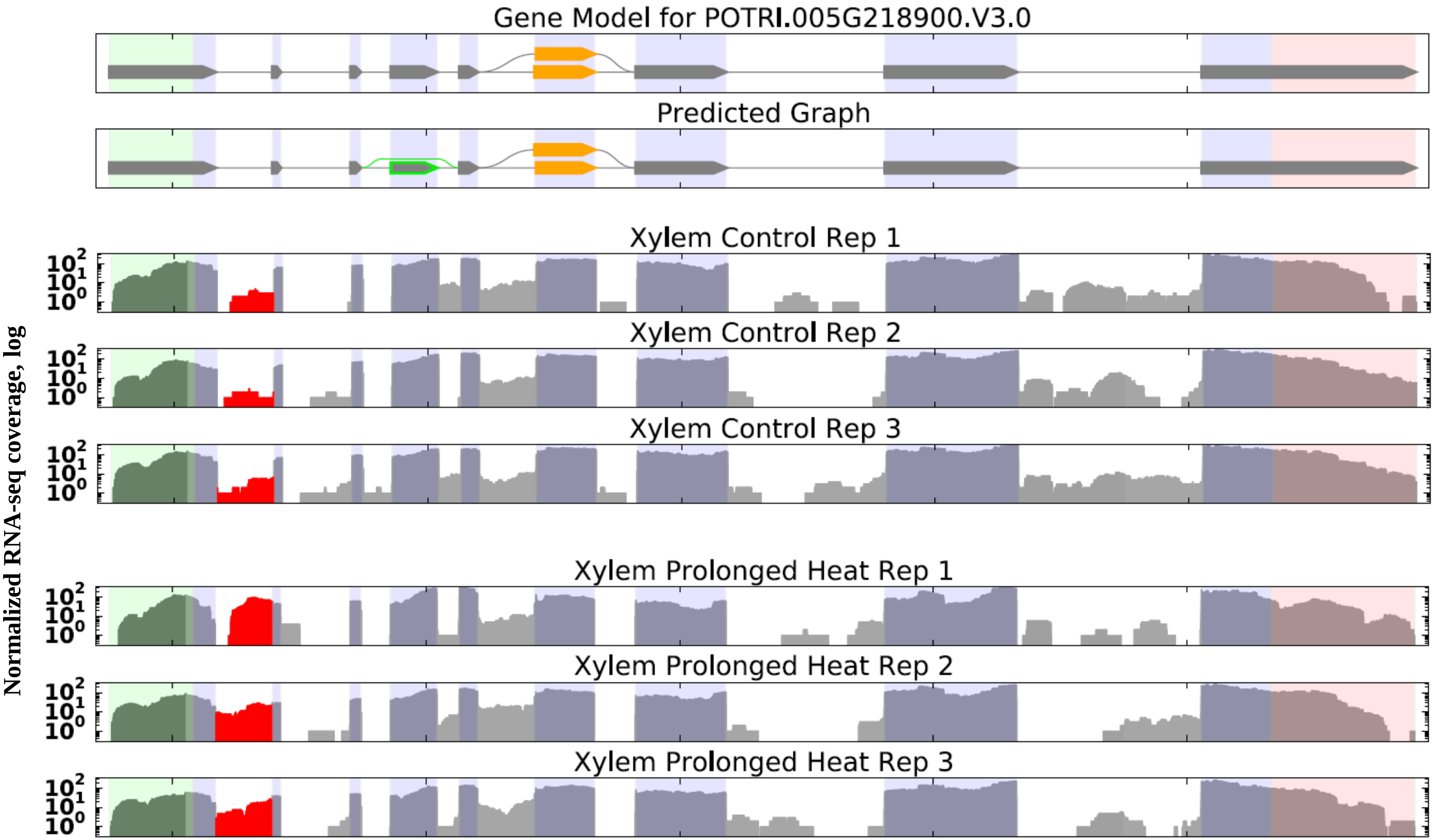

Supplementary File 19. Stress-inducible DIR in mRNA encoding a poplar protein homologous to GALACTURONOSYLTRANSFERASE-LIKE and IRREGULAR XYLEM protein families (*POTRI.005G218900*). Note that retention of the first intron by heat stress occurs in xylem only suggesting a tissue specificity of this event. Graphical output of iDiffIR software showing normalized coverage of a DIR event (depicted in red). Y-axis: the log of normalized intron coverage.
