# Supplementary material for: Abiotic Stresses Modulate Landscape of Poplar Transcriptome via Alternative Splicing, Differential Intron Retention, and Isoform Ratio Switching
Source: Front Plant Sci. 2018 Feb 12;9:5. doi: 10.3389/fpls.2018.00005 (PMC5816337; doi:10.3389/fpls.2018.00005)
Supplement: Supplementary file 2 [file Data_Sheet_2.zip › Supplementary files 17-24/Supplementary File 20.pdf]

Supplementary File 20. Stress-inducible DIR in transcripts of poplar homologs of the key regulators of plant circadian oscillator *ptca1/lhy-l* (A) and *ptgrp7-l* (B). IR events in *ptcca1/lhy* (*circadian clock associated1/late elongated hypocotyl-like*; *POTRI.002G180800*) and *ptgrp7* (*glycine rich protein7-like, POTRI.009G116400*) mRNAs were differentially regulated by the temperature. This observation is consistent with our previous findings of regulation of similar intron retention events in Arabidopsis and *Brachypodium cca1/lhy* homologs (Filichkin *et al*., 2010; Filichkin & Mockler, 2012; Filichkin *et al*., 2015a).

DIR events harbored by the poplar mRNAs of the master circadian regulators

A

circadian clock associated 1/late elongated hypocotyl-like (ptcca1/lhy-like)

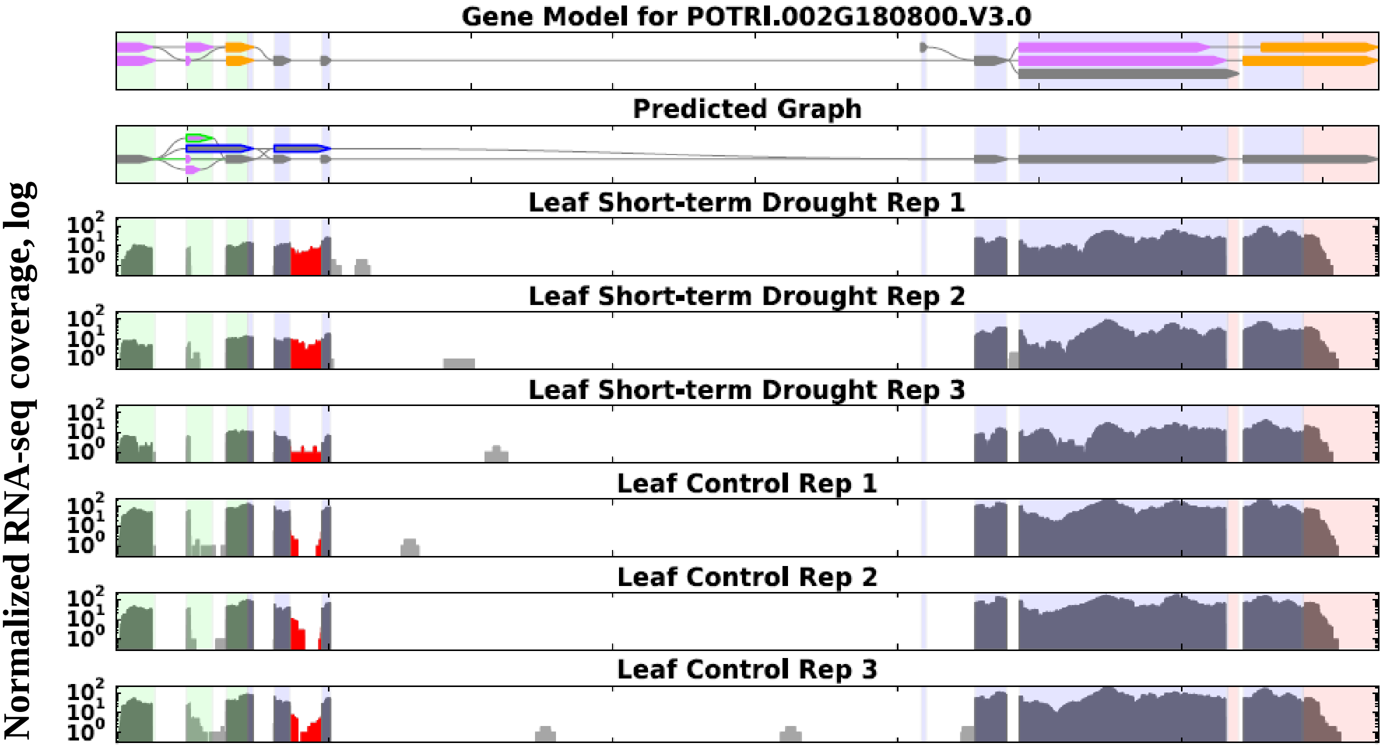

B

glycine rich protein 7-like (ptgrp7-like)

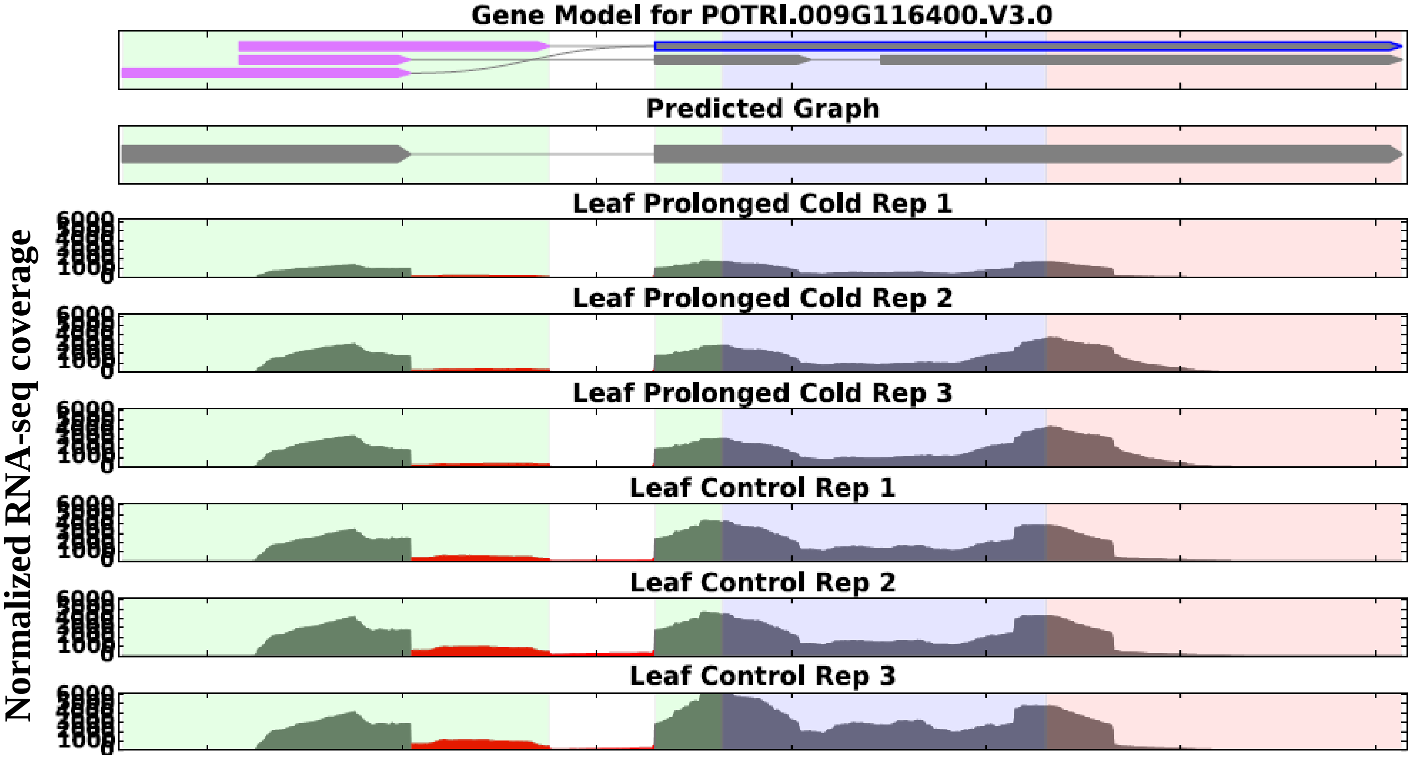

Differentially retained introns
