# Supplementary material for: Abiotic Stresses Modulate Landscape of Poplar Transcriptome via Alternative Splicing, Differential Intron Retention, and Isoform Ratio Switching
Source: Front Plant Sci. 2018 Feb 12;9:5. doi: 10.3389/fpls.2018.00005 (PMC5816337; doi:10.3389/fpls.2018.00005)

Supplementary File 21. An example of stress co-regulated cluster of differential intron retention events (DIRs). Iso-Seq models show mRNAs encoding 40 KDA HEAT SHOCK PROTEIN (*POTRI.010g036200*) (A), SERINE PROTEASE (*POTRI.012G077900*) (B), DYNAMIN (*POTRI.017G041800*) (C), and SAICAR SYNTHASE (*POTRI.017G051500*) (D). The transcript features including all DIRs identified by iDiffIR software (see Fig. 3) consistent with the Iso-Seq models and individual reads. Notably, all DIR events in this cluster are located in the 3’ ends of mRNAs. The retention of introns in all four mRNAs will produce unusually long 3’ untranslated regions potentially targeting these transcripts for degradation by the nonsense mediated mRNA decay pathway.

A

40 kda heat shock protein mRNA (*POTRI.010G036200*)

UNTREATED CONTROL

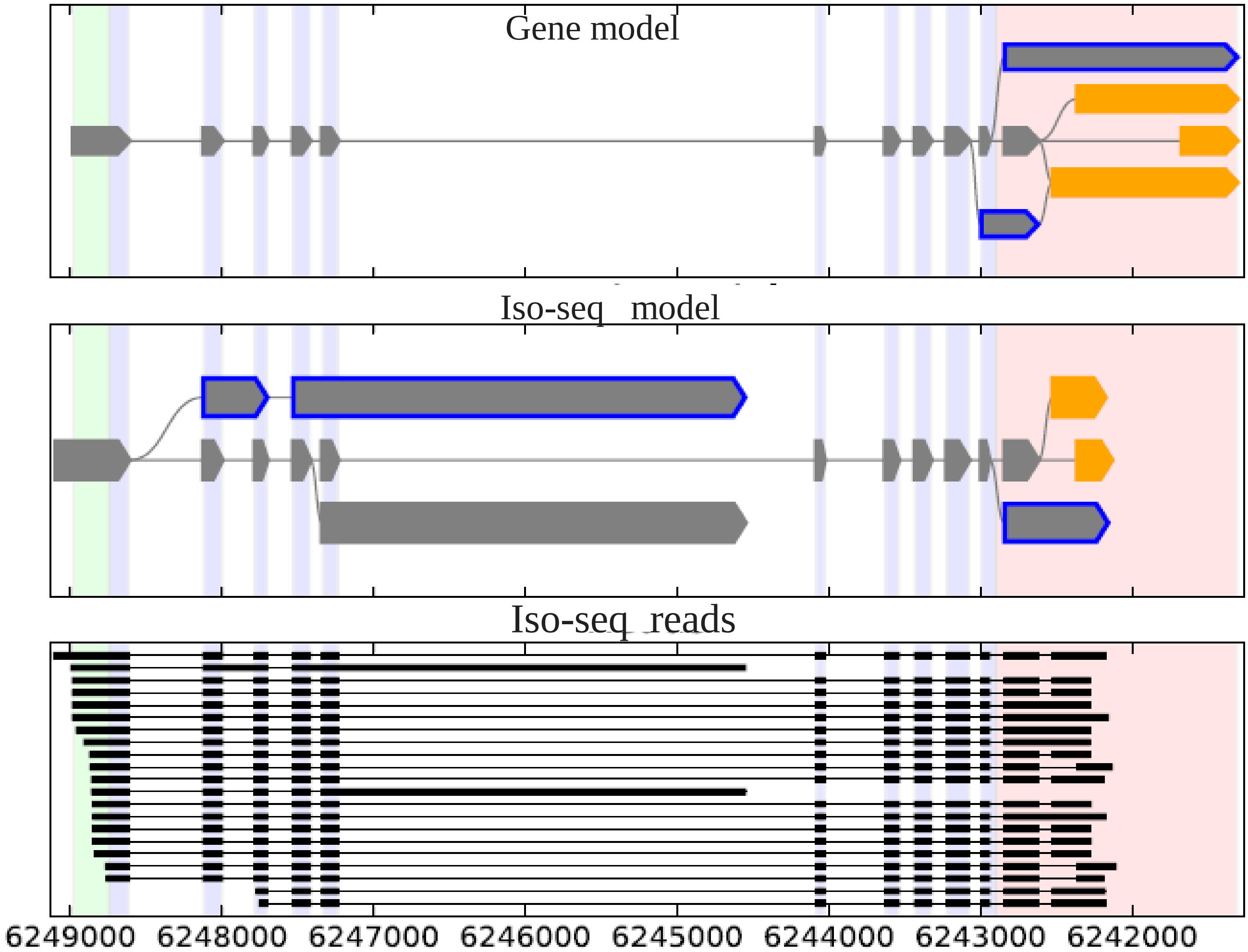

STRESS (combined treatments)

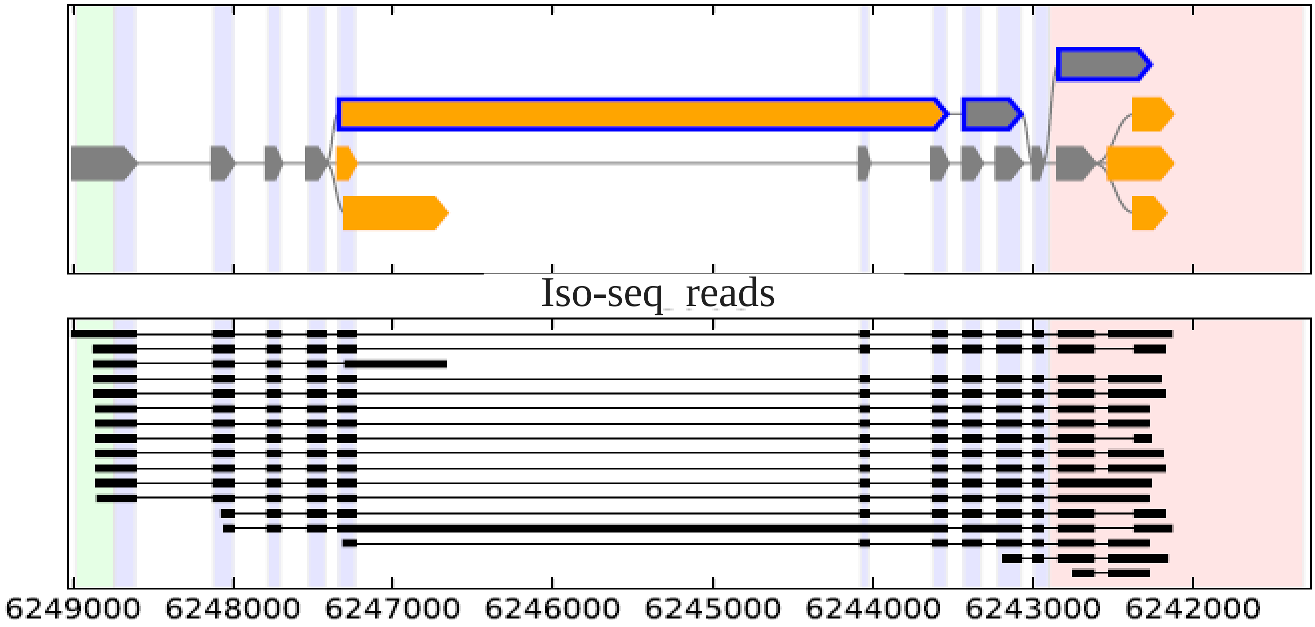

Genomic coordinates (Chromosome 10)

B ***serine protease*** **mRNA (*POTRI.012G077900)***

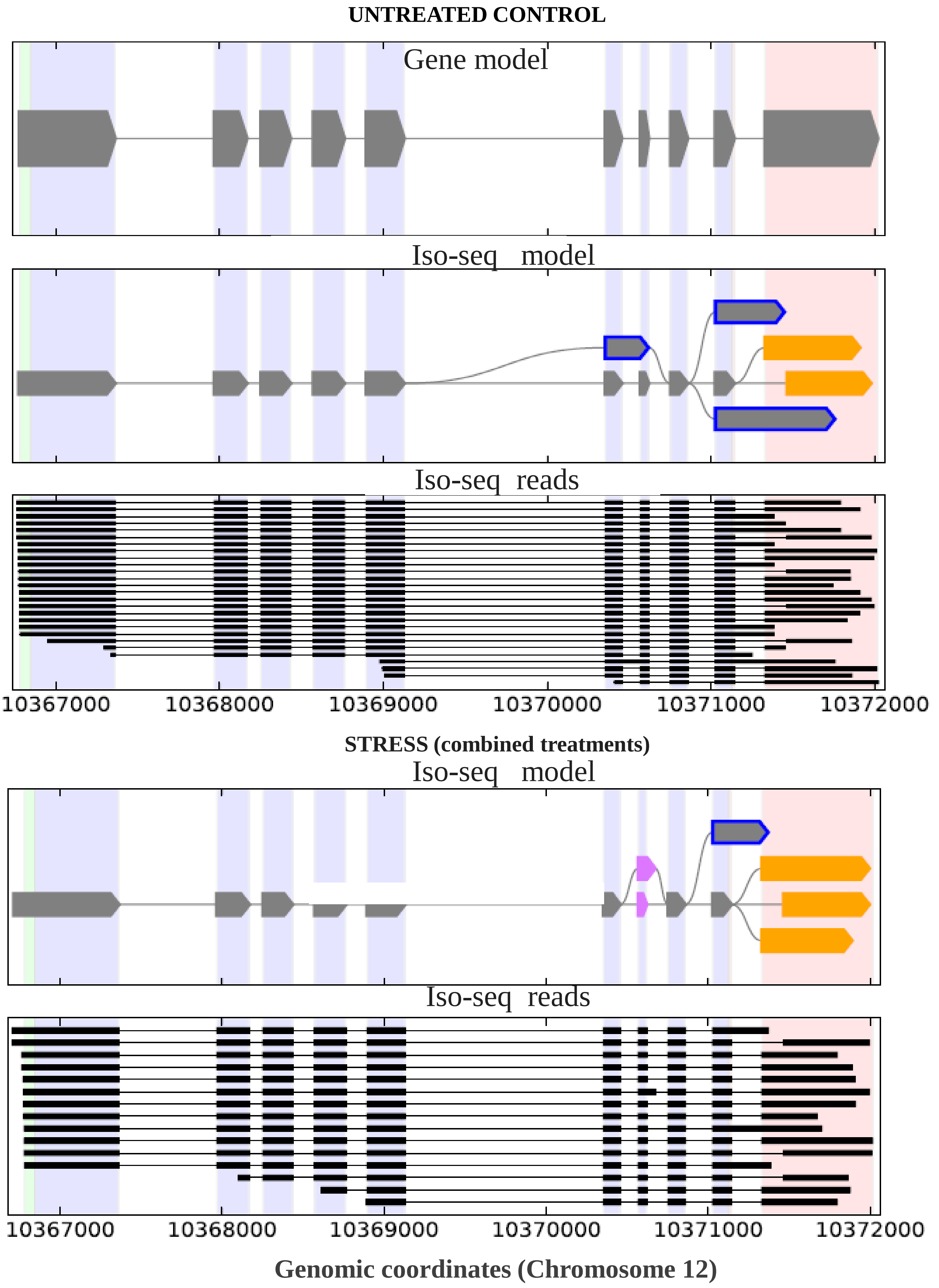

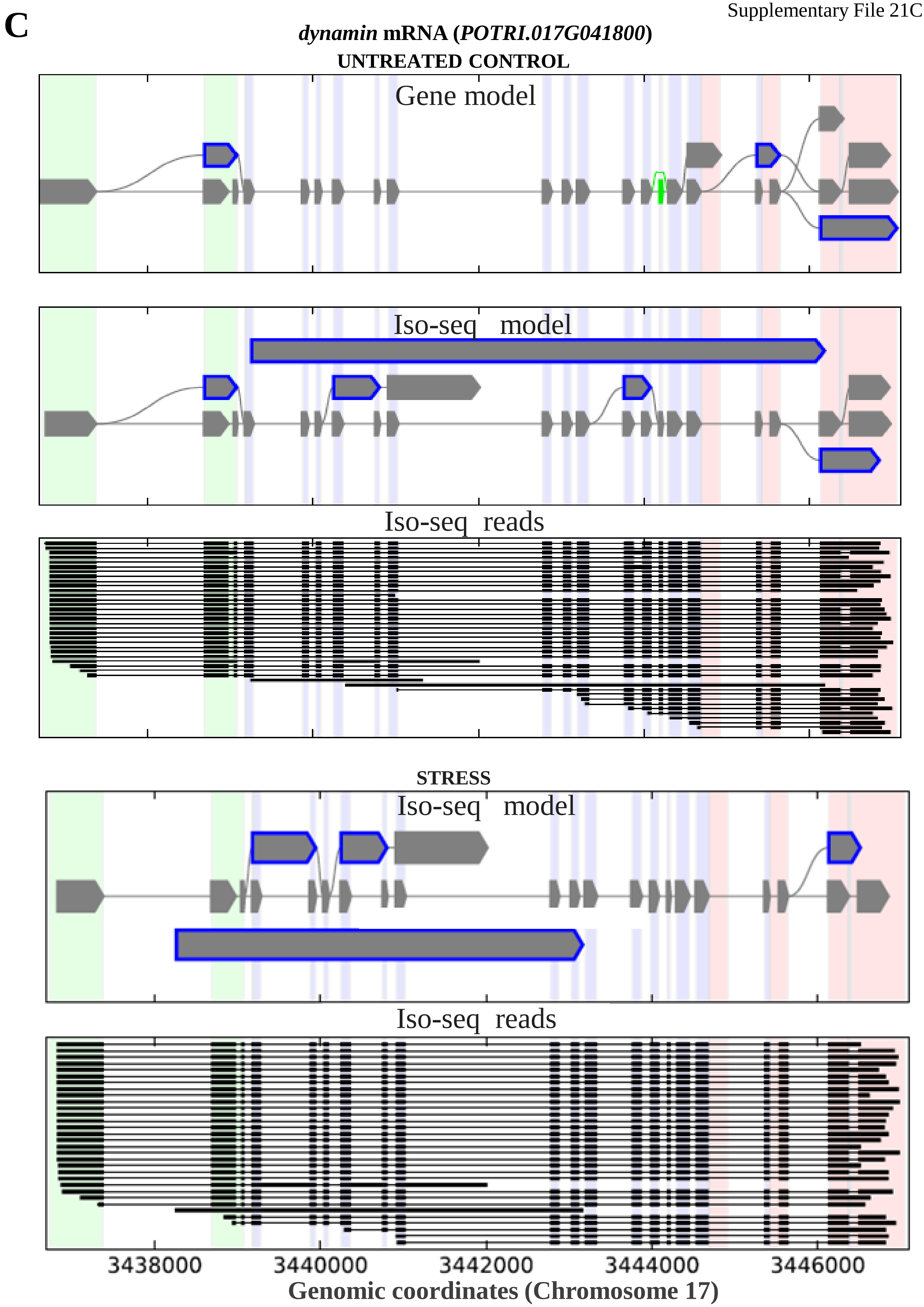
C
dynamin mRNA (POTRI.017G041800)
UNTREATED CONTROL
Gene model
Iso-seq model
Iso-seq reads
STRESS
Iso-seq model
Iso-seq reads
3438000
3440000
3442000
3444000
3446000
Genomic coordinates (Chromosome 17)

D

saicar synthase mRNA (POTRI.017G051500)

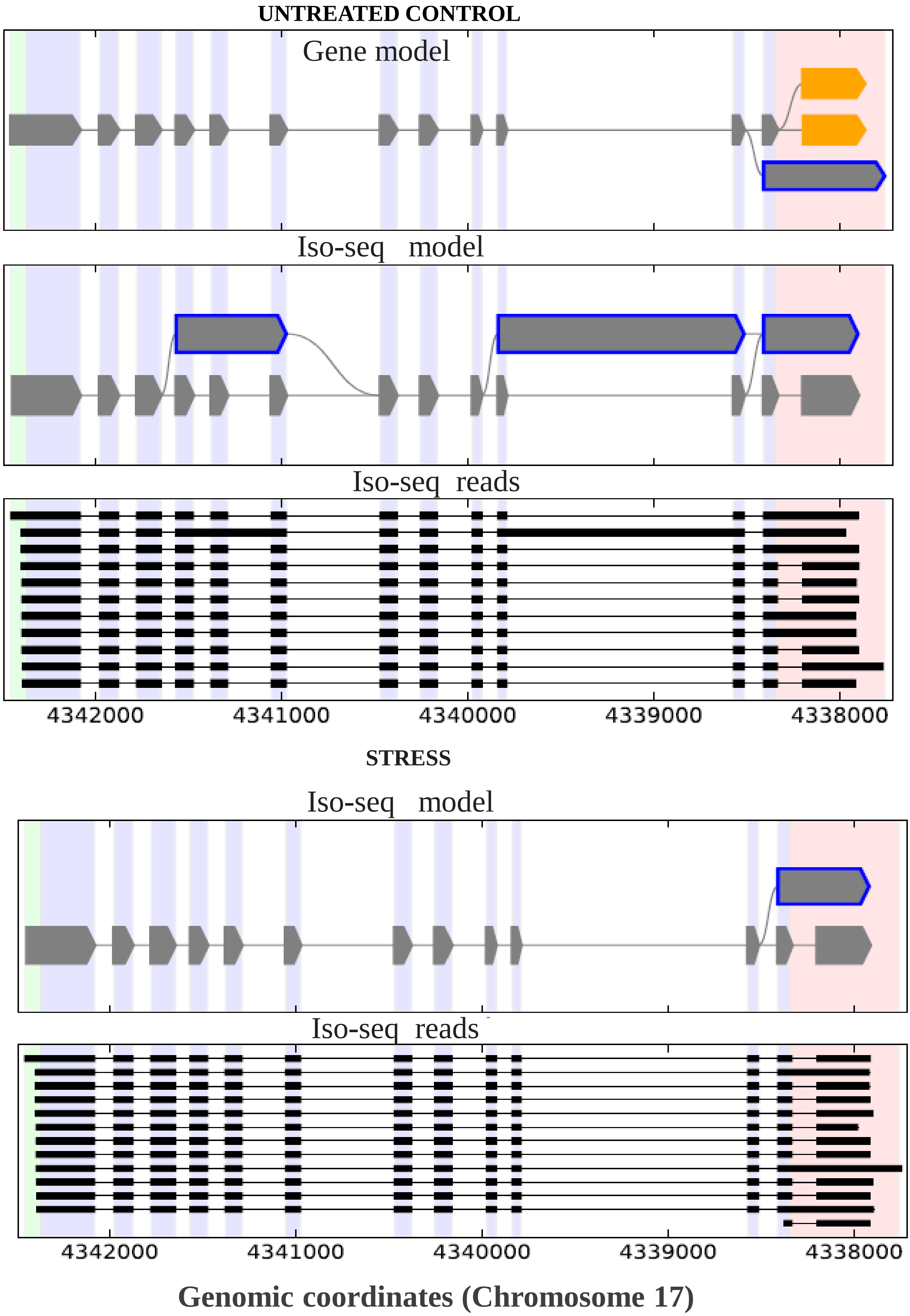

Supplement: Supplementary file 2 [file Data_Sheet_2.zip › Supplementary files 17-24/Supplementary File 21.pdf]
