# Supplementary material for: Abiotic Stresses Modulate Landscape of Poplar Transcriptome via Alternative Splicing, Differential Intron Retention, and Isoform Ratio Switching
Source: Front Plant Sci. 2018 Feb 12;9:5. doi: 10.3389/fpls.2018.00005 (PMC5816337; doi:10.3389/fpls.2018.00005)

Supplementary File 22. Iso-Seq models generally support transcript features (including differentially retained introns) predicted by the iDiffIR software using RNA-Seq data. Iso-Seq-predicted models of poplar *oncogene-related protein 2-like* (*ptocr2-l*) (A) and *general transcription factor* II B (*pttfIIB*) (B) mRNAs. (C) Coverage of *pttfIIB* mRNA by RNA-Seq reads under normal conditions and high/low temperature stresses. Image generated using Poplar Interactome project web site (http://poplar.cgrb.oregonstate.edu/cgi-bin/gb2/gbrowse/poplar/). Retention of the 1st and 6th introns was quantified using droplet digital PCR as described in Fig. 5 and Materials and Methods.

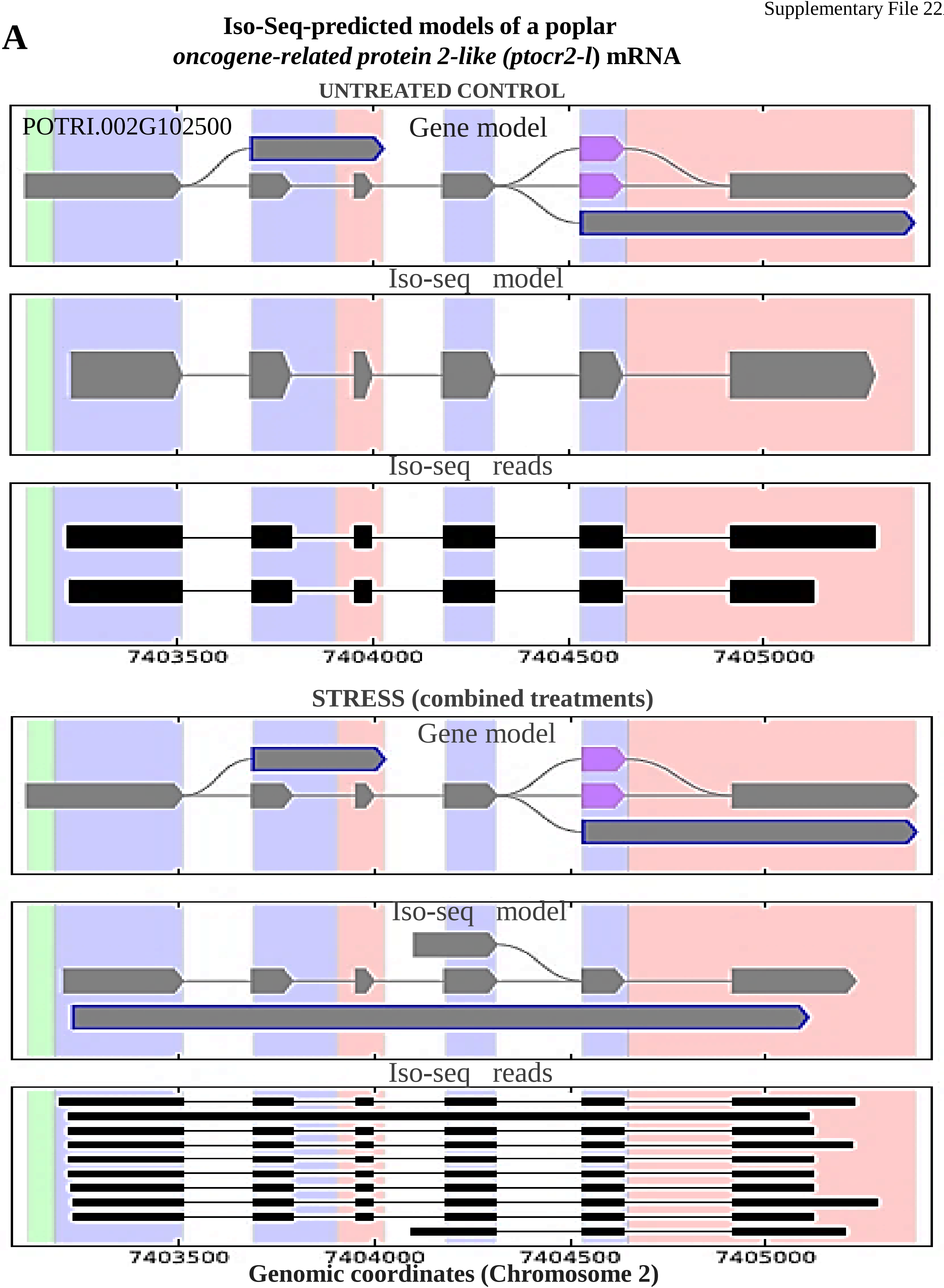
A
Iso-Seq-predicted models of a poplar
oncogene-related protein 2-like (ptocr2-l) mRNA
UNTREATED CONTROL
POTRI.002G102500
Gene model
Iso-seq model
Iso-seq reads
7403500
7404000
7404500
7405000
STRESS (combined treatments)
Gene model
Iso-seq model
Iso-seq reads
7403500
7404000
7404500
7405000
Genomic coordinates (Chromosome 2)

B

Iso-Seq model of poplar mRNA *pttfIIB* encoding GENERAL TRANSCRIPTION FACTOR SUBUNIT B (*POTRI.006G048400*)

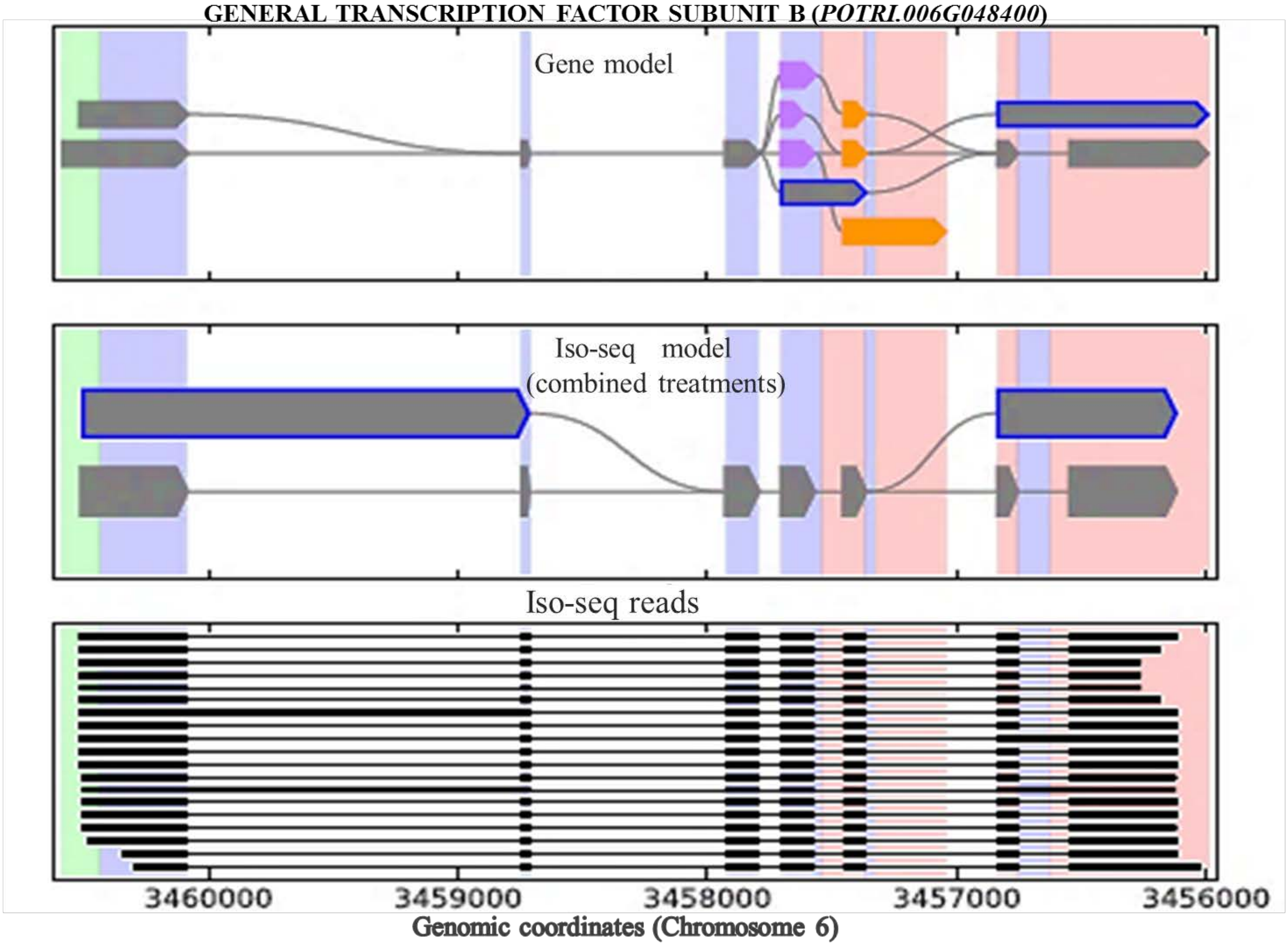

C

RNA-seq coverage and iDiffIR-generted models of poplar *pttfIIB* mRNA (encodes GENERAL TRANSCIPTION INITIATION FACTOR II SUBUNIT B)

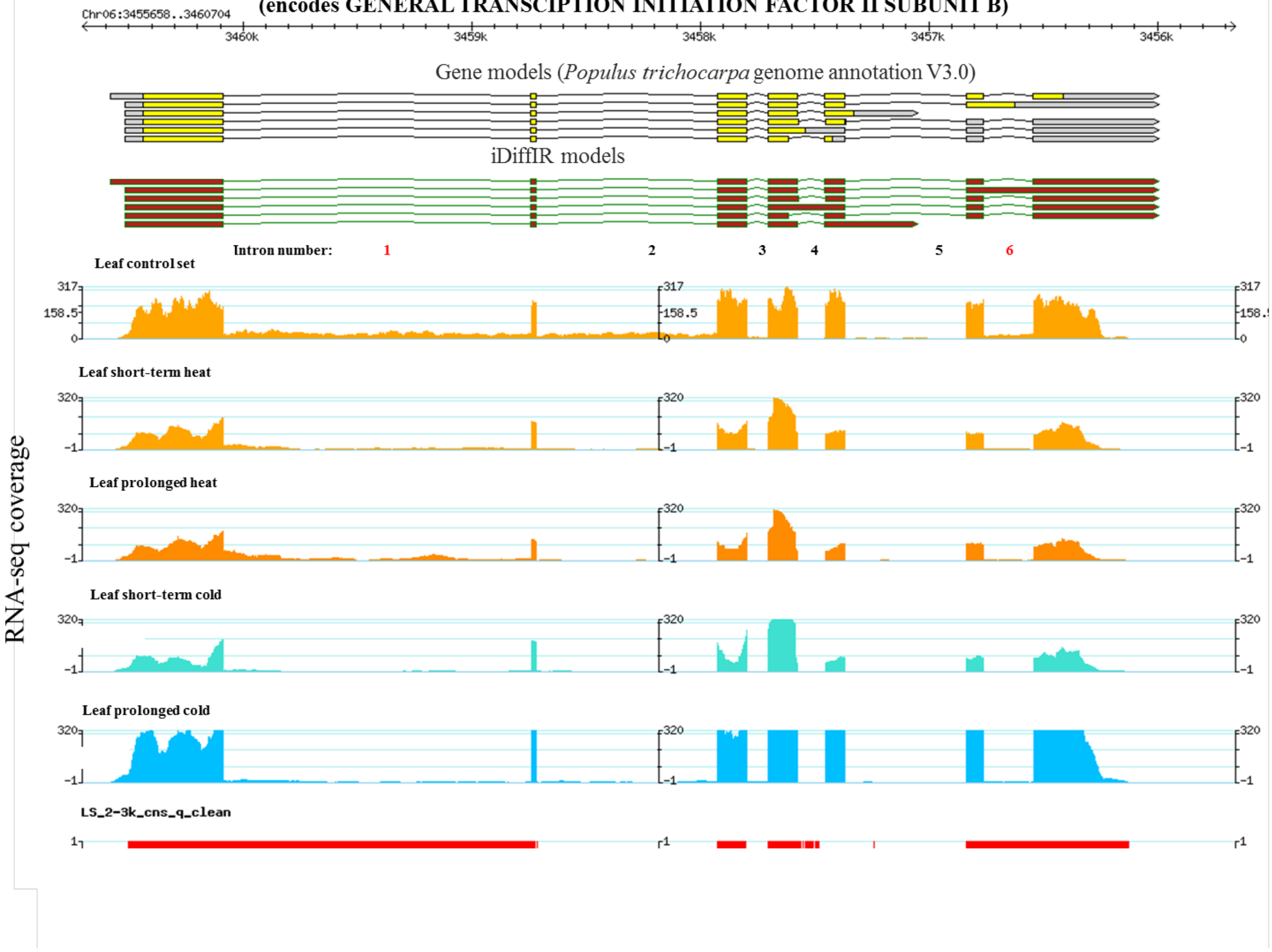

Supplement: Supplementary file 2 [file Data_Sheet_2.zip › Supplementary files 17-24/Supplementary File 22.pdf]
