# Supplementary material for: Abiotic Stresses Modulate Landscape of Poplar Transcriptome via Alternative Splicing, Differential Intron Retention, and Isoform Ratio Switching
Source: Front Plant Sci. 2018 Feb 12;9:5. doi: 10.3389/fpls.2018.00005 (PMC5816337; doi:10.3389/fpls.2018.00005)
Supplement: Supplementary file 3 [file Data_Sheet_3.zip › Supplementary files 25-28/Supplementary File 27.pdf]

Supplementary File 27. Moderate range temperature stress-driven increase of fully spliced *pttfIIB* mRNA (encodes GENERAL TRANSCRIPTION FACTOR II B) is accompanied by moderate increase of the relative ratio of the 1st intron-retaining isoform (I1R). (A) A time course of accumulation of fully spliced *pttfIIB* mRNA copies during increasing (heat stress) or decreasing (cold stress) temperature. (B) A time course of accumulation of the first intron-retaining *pttfIIB* mRNA during high or low temperature stresses. Absolute copy number of each isoform was determined using cDNA from reverse transcription reaction followed by the droplet digital PCR (RT-ddPCR) as described in Materials and Methods. RT-ddPCR of the fully spliced *pttfIIB* mRNA and its first intron-retaining isoform was performed using event-specific primers as described in Materials and Methods. (C) Relative ratio of the first intron-retaining isoform to its fully spliced counterpart mostly decreases with the increase of copy number of spliced mRNA and vice versa. Note that during heat stress treatment segment the level of spliced mRNA shows limited changes whereas relative proportion of I1R isoform increased substantially. Young poplar plants were heat treated (42°C) for 24 hours followed by the transition to cold temperature (4°C) for another 24 hours as described in Materials and Methods. Diagram at the bottom shows fluctuations of temperature during the time course of treatment. Each time point represents two hours.

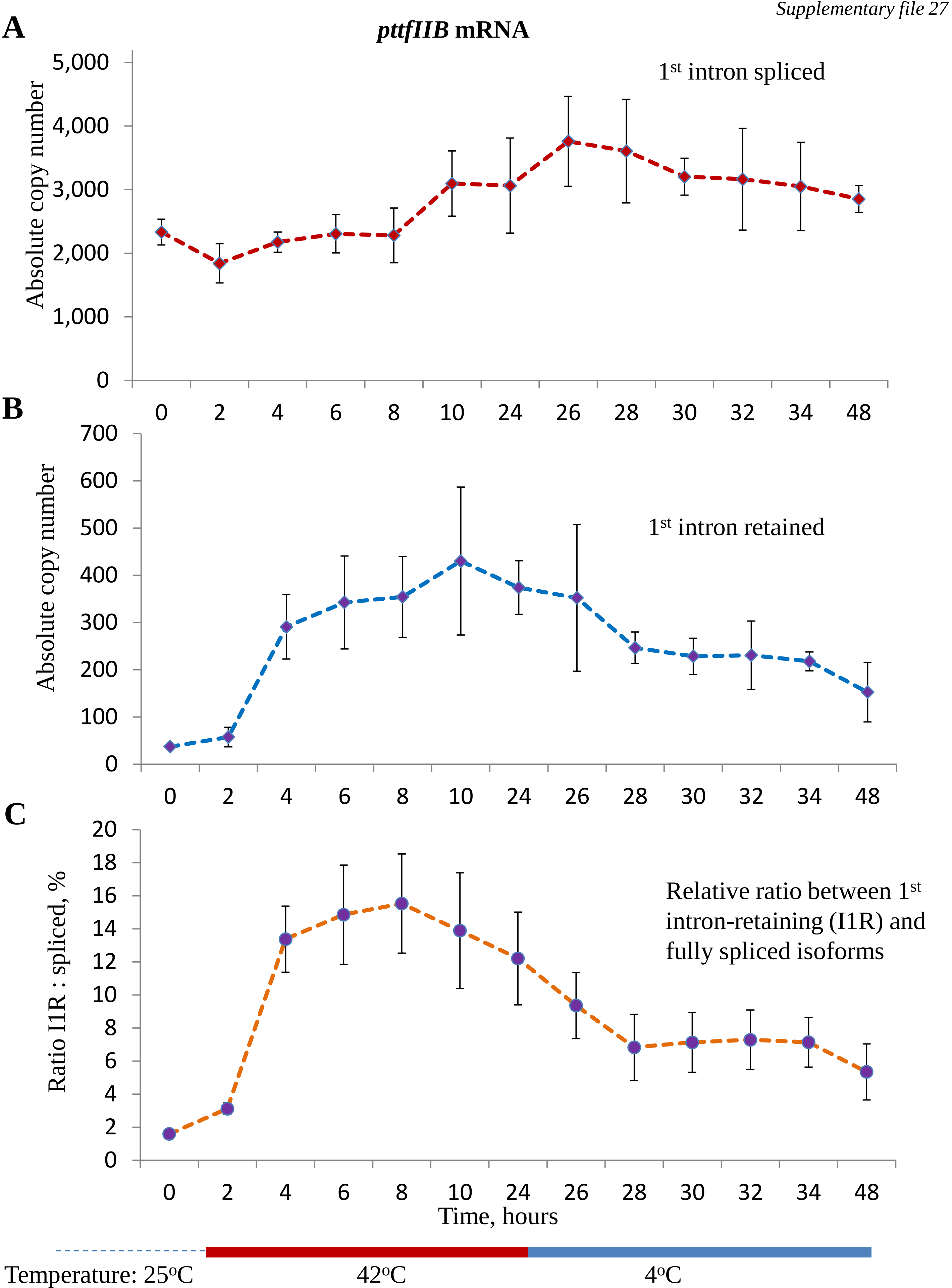
A
pttfIIB mRNA
1st intron spliced
Absolute copy number
5,000
4,000
3,000
2,000
1,000
0
0 2 4 6 8 10 24 26 28 30 32 34 48
B
1st intron retained
Absolute copy number
700
600
500
400
300
200
100
0
0 2 4 6 8 10 24 26 28 30 32 34 48
C
Relative ratio between 1st intron-retaining (I1R) and fully spliced isoforms
Ratio I1R : spliced, %
20
18
16
14
12
10
8
6
4
2
0
0 2 4 6 8 10 24 26 28 30 32 34 48
Time, hours
Temperature: 25oC
42oC
4oC
