# Supplementary material for: Abiotic Stresses Modulate Landscape of Poplar Transcriptome via Alternative Splicing, Differential Intron Retention, and Isoform Ratio Switching
Source: Front Plant Sci. 2018 Feb 12;9:5. doi: 10.3389/fpls.2018.00005 (PMC5816337; doi:10.3389/fpls.2018.00005)
Supplement: Supplementary file 3 [file Data_Sheet_3.zip › Supplementary files 25-28/Supplementary File 28.pdf]

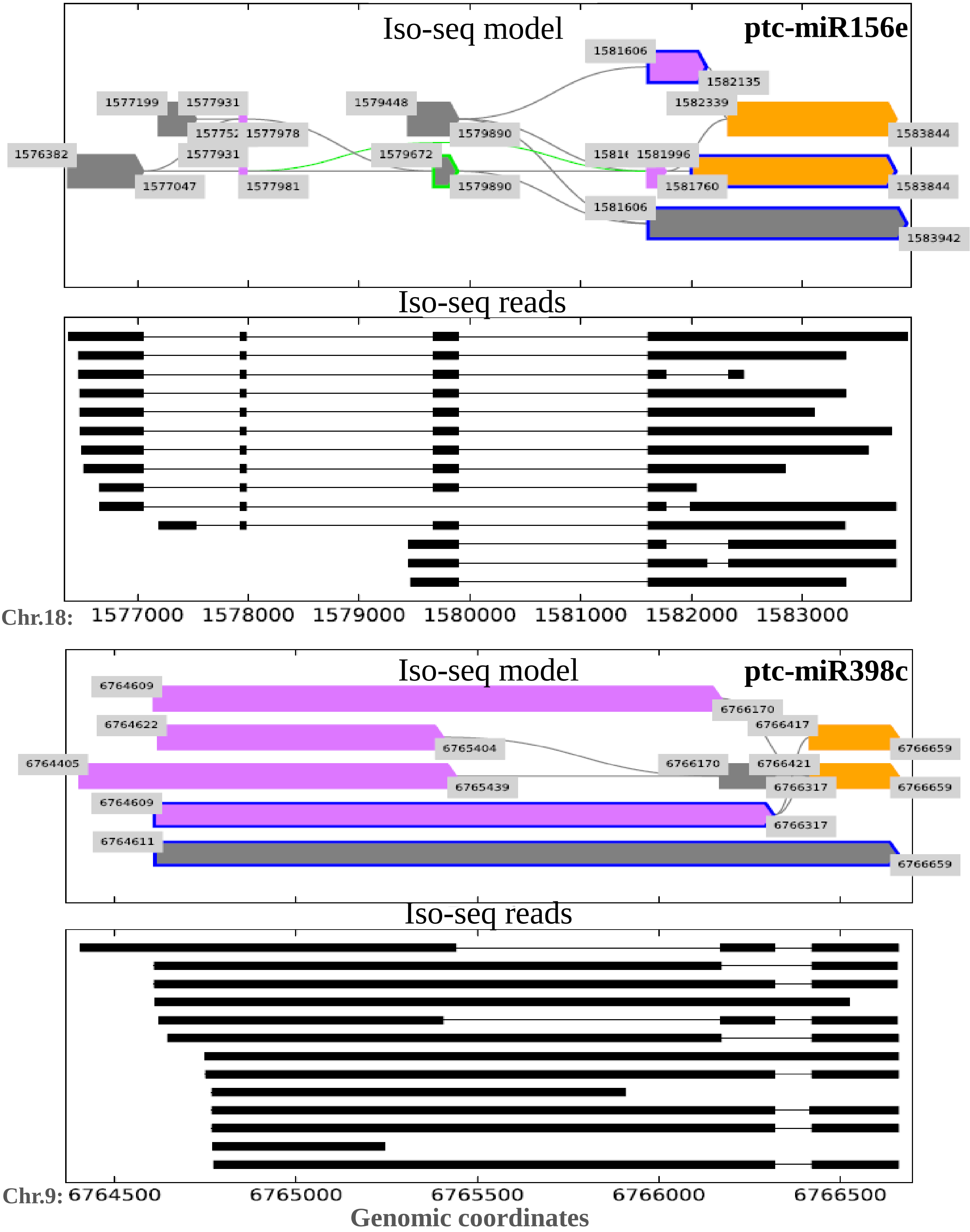

Supplementary File 28. Examples of extensive alternative splicing and multiple intron retention events in primary transcripts of poplar miRNAs ptc-miR156e and ptc-miR398c. Numbers in gray boxes indicate genomic coordinates of splice junctions.
